# Supplementary material for: Structural insights into multiplexed pharmacological actions of tirzepatide and peptide 20 at the GIP, GLP-1 or glucagon receptors
Source: Nat Commun. 2022 Feb 25;13:1057. doi: 10.1038/s41467-022-28683-0 (PMC8881610; doi:10.1038/s41467-022-28683-0)
Supplement: Supplementary file 1 — Supplementary Information [file 41467_2022_28683_MOESM1_ESM.pdf]

Supplementary Information

Structural insights into multiplexed pharmacological actions of tirzepatide and peptide 20 at the GIP, GLP-1 or glucagon receptors

Brief description of what this file includes:

Supplementary Fig. 1 | Principles of combinatorial agonism to synergize metabolic actions and maximize therapeutic benefits.

Supplementary Fig. 2 | Receptor constructs for structure determination.

Supplementary Fig. 3 | Purification and characterization of the tirzepatide–GIPR/GLP-1R–G_s–Nb35 complexes and non-acylated tirzepatide–GIPR/GLP-1R–G_s–Nb35 complexes.

Supplementary Fig. 4 | Purification and characterization of the peptide 20–GIPR/GLP-1R/GCGR–G_s–Nb35 complexes.

Supplementary Fig. 5 | Cryo-EM data processing and validation.

Supplementary Fig. 6 | Near-atomic resolution model of the complexes in the cryo-EM density maps.

Supplementary Fig. 7 | Structural comparison of tirzepatide and non-acylated tirzepatide bound GIPR and GLP-1R.

Supplementary Fig. 8 | Molecular dynamics (MD) simulation of GLP-1R bound by tirzepatide.

Supplementary Fig. 9 | Effect of receptor mutation on peptide 20-induced cAMP accumulation and receptor binding affinity.

Supplementary Fig. 10 | Molecular dynamics (MD) simulation of GLP-1R bound by peptide 20.

Supplementary Fig. 11 | Conformational changes upon GCGR activation.

Supplementary Fig. 12 | Gating strategy of cell surface expression assay.

Supplementary Table 1 | Mono-, dual and triple agonists at GLP-1R, GCGR or GIPR that entered into clinical development.

Supplementary Table 2 | Cryo-EM data collection, refinement and validation statistics.

Supplementary Table 3 | cAMP signaling and receptor binding profiles of tirzepatide, non-acylated tirzepatide and peptide 20 at GIPR or GLP-1R.

Supplementary Table 4 | Interaction between tirzepatide and GIPR or GLP-1R.

Supplementary Table 5 | Interaction between peptide 20, GIPR, GLP-1R and GCGR.

Supplementary Table 6 | cAMP signaling profiles of endogenous agonists, multi-targeting agonists and approved GLP-1 analogs at GIPR, GLP-1R and GCGR.

Supplementary Table 7 | Effects of the ligand-binding pocket residue mutation on tirzepatide-induced cAMP responses and receptor binding profiles.

Supplementary Table 8 | Effects of the ligand-binding pocket residue mutation on peptide 20-induced cAMP signaling and receptor binding profiles at GIPR, GLP-1R and GCGR.

Supplementary Table 9 | Effects of ligand-binding pocket residue mutation on receptor expression.

Supplementary Table 10 | Signaling profiles of mono- and triple agonists at GIPR, GLP-1R and GCGR.

Supplementary Table 11 | Signaling profiles of mono- and dual agonists at GIPR and GLP-1R.

Supplementary Table 12 | Primers used in this study, related to Figs. 3, 5, Supplementary Figs. 2, 3, 4, 9 and Supplementary Tables 3, 6, 7, 8, 9, 10, 11.

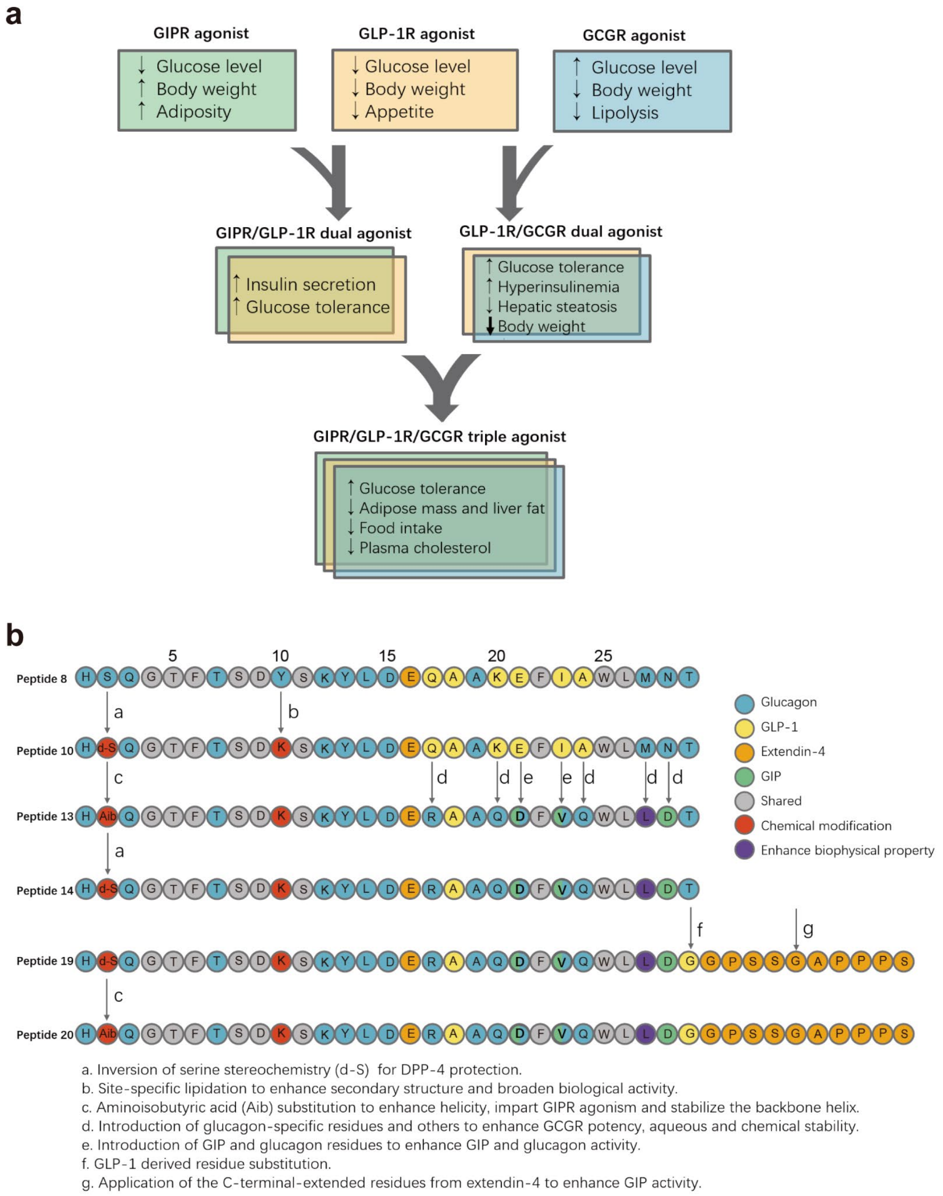

Supplementary Fig. 1 | Principles of combinatorial agonism to synergize metabolic actions and maximize therapeutic benefits. a, Schematic representation of the therapeutic advantages of dual and triple agonists targeting the human glucose-dependent insulinotropic polypeptide (GIP), glucagon-like peptide-1 (GLP-1) and glucagon (GCG) receptors (GIPR, GLP-1R and GCGR, respectively). GLP-1R agonists are used to treat type 2 diabetes and obesity because of their ability to promote satiety and insulin secretion. Their effect on weight loss could be complemented by that of glucagon on lipolysis and thermogenesis, leading to a series of GLP-1R/GCGR dual agonists (e.g., peptide 8) based on the sequence of GCG. Subsequently, GIPR agonism was added to GLP-1R agonists to enhance the glycemic benefits of GLP-1 resulting in a new series of dual agonists (e.g., tirzepatide) that improved insulin secretion and glucose tolerance while reducing adverse events of the monotherapy. Given the enhanced performance of both dual agonists in the treatment of obesity and T2D, as well as the structural similarity among the three peptides, Multi-targeting GIPR/GLP-1R/ GCGR triple agonists (e.g., peptide 20) were developed to combine the strength of both types of dual agonists. **b,** Evolutionary pathway towards a highly potent and balanced unimolecular triple agonist (peptide 20) for GIPR, GLP-1R and GCGR. The modifications and their actions on combinatorial agonism are explained in the bottom.

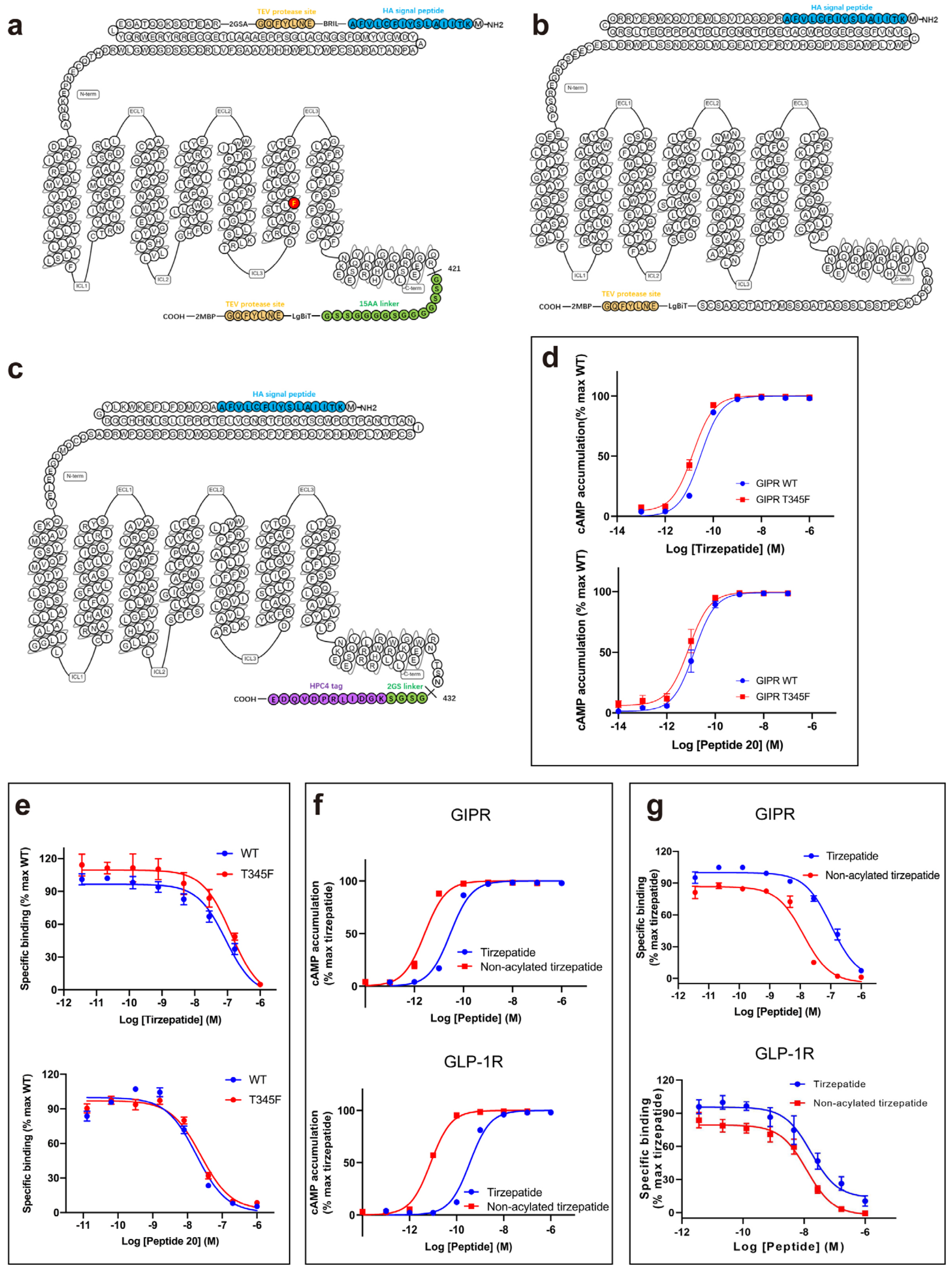

Supplementary Fig. 2 | Receptor constructs for structure determination. a-c, Schematic diagrams of receptor constructs used for structure determination: GIPR construct (**a**), GLP-1R construct (**b**) and GCGR construct (**c**). **d,** Effects of GIPR T345F on tirzepatide (top) and peptide 20 (bottom)-induced cAMP accumulation. Data shown are means ± S.E.M. of at least three independent experiments (*n* = 3-9) performed in quadruplicate. **e,** Effects of GIPR T345F on receptor binding affinities of tirzepatide (top) and peptide 20 (bottom). Data shown are means ± S.E.M. of at least three independent experiments (*n* = 3-5) performed in duplicate. **f,** Effects of tirzepatide acylation on GIPR (top) and GLP-1R (bottom)-mediated cAMP accumulation. Data shown are means ± S.E.M. of three independent experiments (*n* = 3) performed in quadruplicate. **g,** Effects of tirzepatide acylation on receptor binding affinities with GIPR (top) and GLP-1R (bottom). Data shown are means ± S.E.M. of at least four independent experiments (*n* = 4-5) performed in quadruplicate. cAMP accumulation and binding data were normalized to the maximum response of wild-type (WT) or tirzepatide and concentration-response curves were analyzed using a three-parameter logistic equation. Source data are provided as a Source Data file.

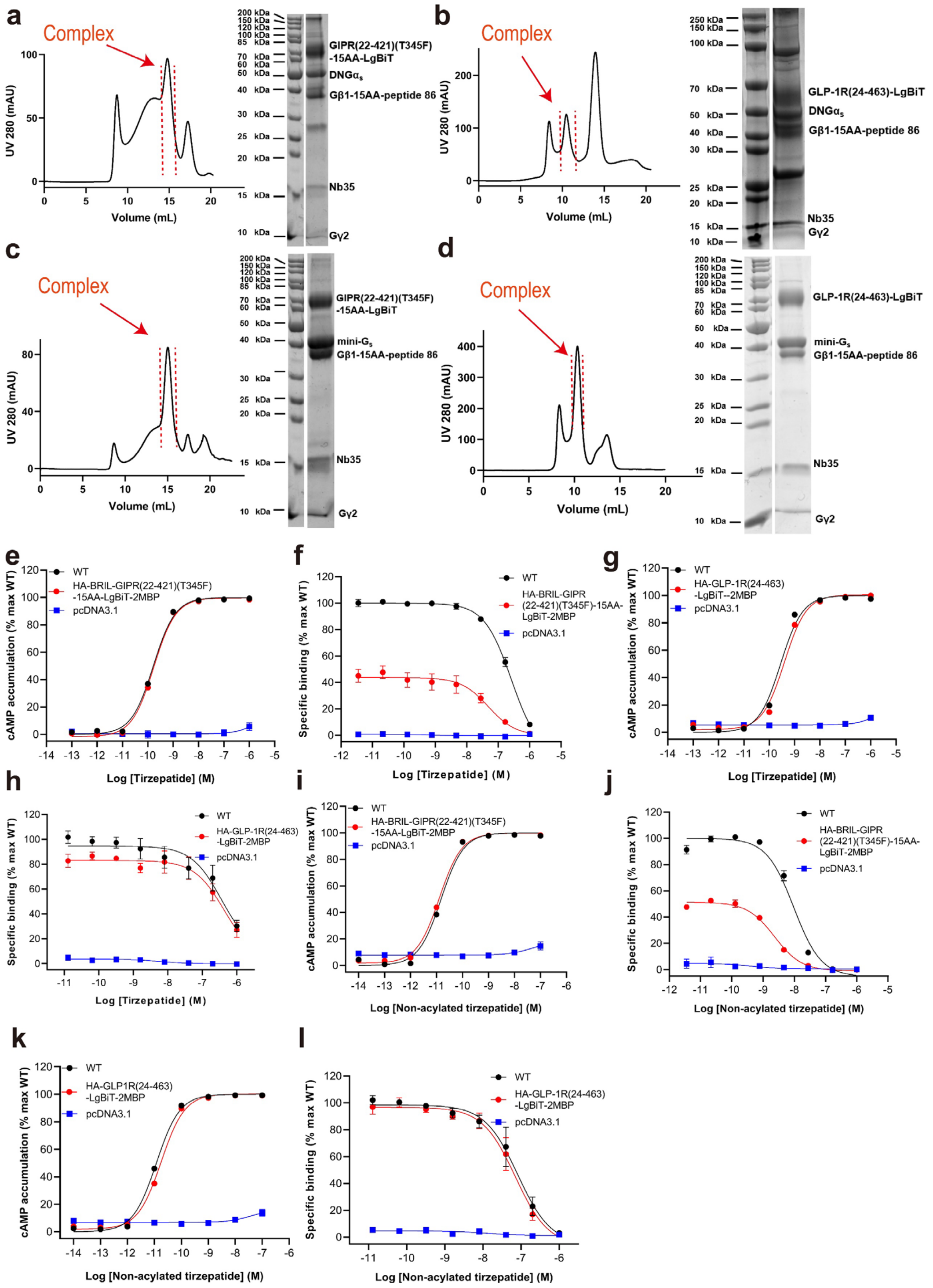

Supplementary Fig. 3 | Purification and characterization of the tirzepatide–GIPR/GLP-1R–G$_s$–Nb35 complexes and non-acylated tirzepatide–GIPR/GLP-1R–G$_s$–Nb35 complexes. a, Size-exclusion chromatography on Superose 6 Increase 10/300GL and SDS-PAGE of the tirzepatide–GIPR–G$_s$–Nb35 complex. **b,** Size-exclusion chromatography on Superdex 200 Increase 10/300GL and SDS-PAGE of the tirzepatide–GLP-1R–G$_s$–Nb35 complex. **c,** Size-exclusion chromatography on Superose 6 Increase 10/300GL and SDS-PAGE of the non-acylated tirzepatide–GIPR-mini–G$_s$–Nb35 complex. **d,** Size-exclusion chromatography on Superdex 200 Increase 10/300GL and SDS-PAGE of the non-acylated tirzepatide–GLP-1R–mini-G$_s$–Nb35 complex. These experiments (**a-d**) were repeated independently twice with similar results. **e,** cAMP responses following tirzepatide stimulation in HEK293T cells transfected with wild-type (WT) or modified GIPR constructs. Data shown are means ± S.E.M. of three independent experiments (n = 3) performed in quadruplicate. **f,** Binding of tirzepatide to the full-length or modified GIPR in competition with ^{125}I-GIP$_{1\text{-}42}$. Data shown

are means ± S.E.M. of three independent experiments (n = 3) performed in duplicate. **g,** cAMP responses following tirzepatide stimulation in HEK293T cells transfected with WT or modified GLP-1R constructs. Data shown are means ± S.E.M. of three independent experiments (n = 3) performed in quadruplicate. **h,** Binding of tirzepatide to the full-length or modified GLP-1R in competition with ^{125}I-GLP-$1_{(7\text{-}36)}NH_2$. Data shown are means ± S.E.M. of four independent experiments (n = 4) performed in duplicate. **i,** cAMP responses following non-acylated tirzepatide stimulation in HEK293T cells transfected with WT or modified GIPR constructs. Data shown are means ± S.E.M. of three independent experiments (n = 3) performed in quadruplicate. **j,** Binding of non-acylated tirzepatide to the full-length or modified GIPR in competition with ^{125}I-$GIP_{1\text{-}42}$. Data shown are means ± S.E.M. of three independent experiments (n = 3) performed in duplicate. **k,** cAMP responses following non-acylated tirzepatide stimulation in HEK293T cells transfected with WT or modified GLP-1R constructs. Data shown are means ± S.E.M. of three independent experiments (n = 3) performed in quadruplicate. **l,** Binding of non-acylated tirzepatide to the full-length or modified GLP-1R in competition with ^{125}I-GLP-$1_{(7\text{-}36)}NH_2$. Data shown are means ± S.E.M. of four independent experiments (n = 4) performed in duplicate. Signals were normalized to the maximum response of the WT and dose-response curves were analyzed using a three-parameter logistic equation. Whole cell binding assay was performed in CHO-K1 cells. Binding data were analyzed using a three-parameter logistic equation to determine pIC_{50} and span values. Source data are provided as a Source Data file.

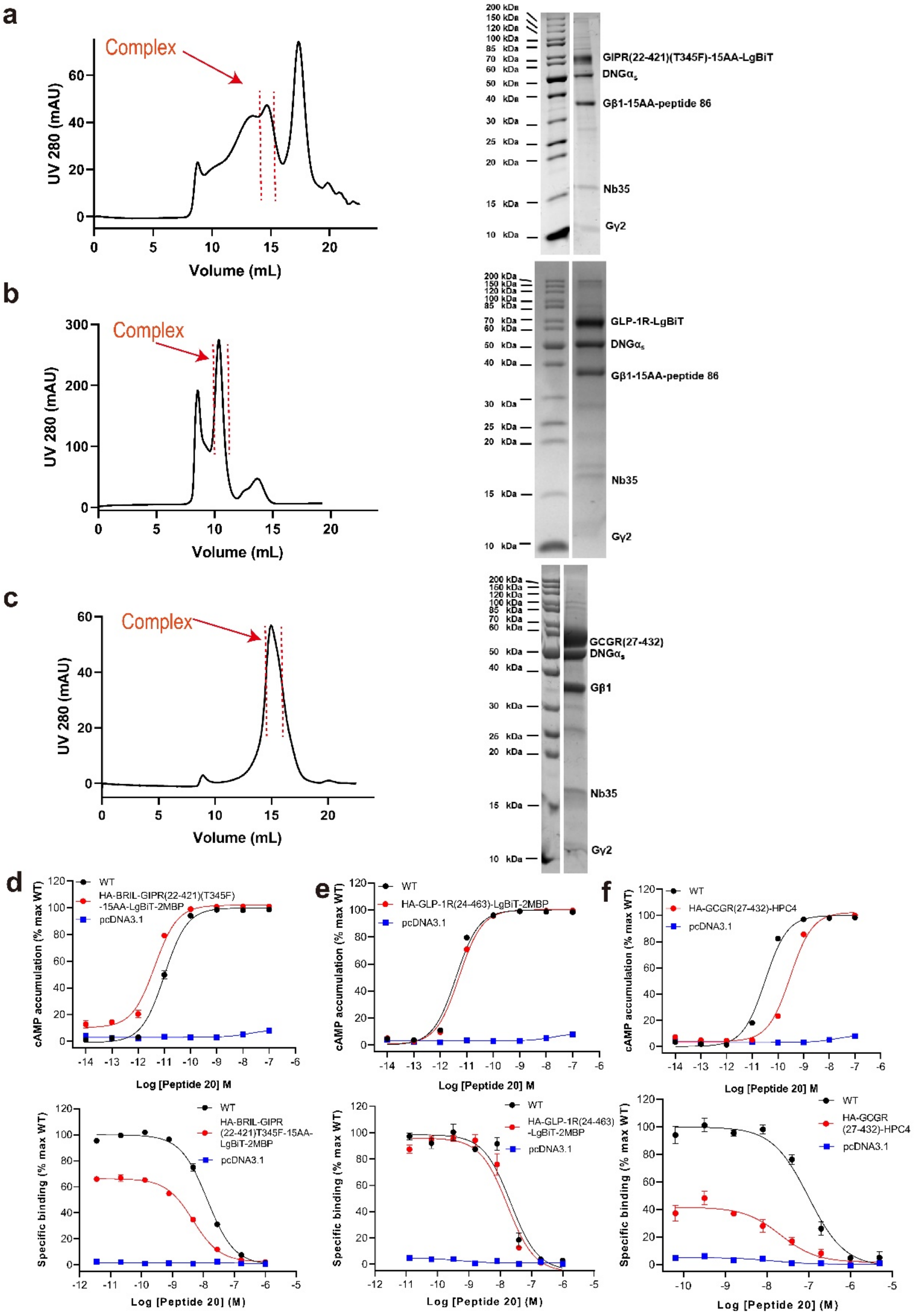

Supplementary Fig. 4 | Purification and characterization of the peptide 20–GIPR/GLP-1R/GCGR–G_s–Nb35 complexes. a, Size-exclusion chromatography on Superose 6 Increase 10/300GL and SDS-PAGE of the peptide 20–GIPR–G_s–Nb35 complex. **b,** Size-exclusion chromatography on Superdex 200 Increase 10/300GL and SDS-PAGE of the peptide 20–GLP-1R–G_s–Nb35 complex. **c,** Size-exclusion chromatography on Superose 6 Increase 10/300GL and SDS-PAGE of the peptide 20–GCGR–G_s–Nb35 complex. These experiments (**a-c**) were repeated independently twice with similar results. **d,** Top, cAMP responses following peptide 20 stimulation in HEK293T cells transfected with wild-type (WT) or modified GIPR constructs. Bottom, binding of peptide 20 to the full-length or modified GIPR in competition with ^{125}I-GIP$_{1\text{-}42}$. Data shown are means ± S.E.M. of three independent experiments (*n* = 3) performed in quadruplicate

(cAMP accumulation) or duplicate (receptor binding assay). **e,** Top, cAMP responses following peptide 20 stimulation in HEK293T cells transfected with WT or modified GLP-1R constructs. Bottom, binding of peptide 20 to the full-length or modified GLP-1R in competition with ^{125}I-GLP-$1_{(7-36)}$$NH_2$. Data shown are means ± S.E.M. of at least three independent experiments (n = 3-4) performed in quadruplicate (cAMP accumulation) or duplicate (receptor binding assay). **f,** Top, cAMP responses following peptide 20 stimulation in HEK293T cells transfected with WT or modified GCGR constructs. Bottom, binding of peptide 20 to the full-length or modified GCGR in competition with ^{125}I-GCG. Data shown are means ± S.E.M. of three independent experiments (n = 3) performed in quadruplicate (cAMP accumulation) or duplicate (receptor binding assay). Signals were normalized to the maximum response of the WT and dose-response curves were analyzed using a three-parameter logistic equation. Whole cell binding assay was performed in CHO-K1 cells. Binding data were analyzed using a three-parameter logistic equation to determine pIC_{50} and span values. Source data are provided as a Source Data file.

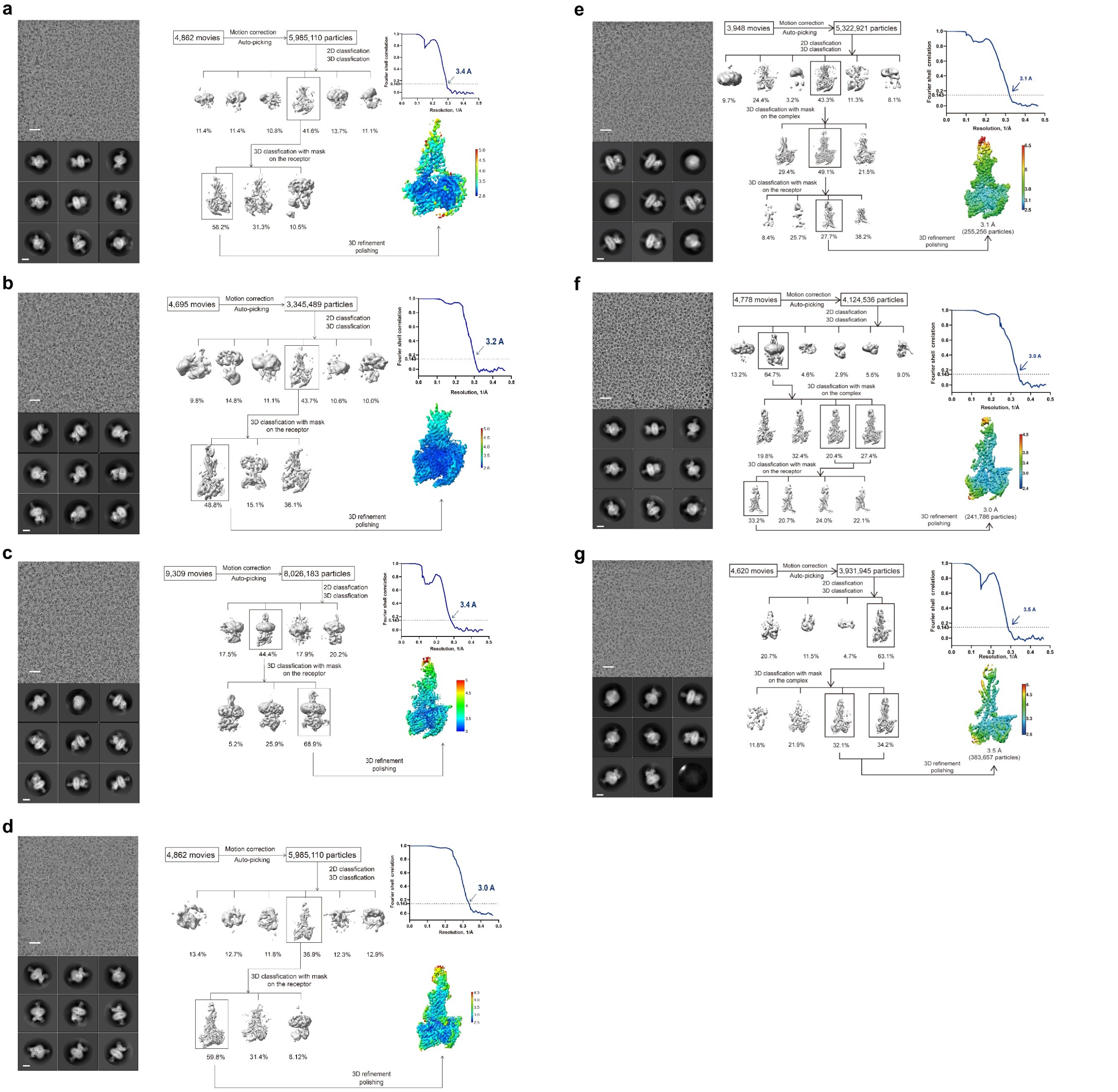

Supplementary Fig. 5 | Cryo-EM data processing and validation. a, Tirzepatide–GIPR–G$_s$ complex: top left, representative cryo-EM micrograph (scale bar: 40 nm) and two-dimensional class averages (scale bar: 5 nm); top right, flow chart of cryo-EM data processing; bottom left, local resolution distribution map of the complex with the ECD and Gold-standard Fourier shell correlation (FSC) curves of overall refined receptor; bottom right, local resolution distribution map of the complex without the ECD and FSC curves of overall refined receptor. **b,** Non-acylated tirzepatide–GIPR–G$_s$ complex: left, representative cryo-EM micrograph (scale bar: 40 nm) and two-dimensional class averages (scale bar: 5 nm); middle, flow chart of cryo-EM data processing; right, local resolution distribution map of the complex and FSC curves of overall refined receptor. The experiments were conducted twice independently with similar results. **c,** Tirzepatide–GLP-R–G$_s$ complex: left, representative cryo-EM micrograph (scale bar: 40 nm) and two-dimensional class averages (scale bar: 5 nm); middle, flow chart of cryo-EM data processing; right, local resolution distribution map of the complex and FSC curves of overall refined receptor. **d,** Non-acylated tirzepatide–GLP-1R–G$_s$ complex: left, representative cryo-EM micrograph (scale bar: 40 nm) and two-dimensional class averages (scale bar: 5 nm); middle, flow chart of cryo-EM data processing; right, local resolution distribution map of the complex and FSC curves of overall refined receptor. The experiments were performed twice independently with similar results. **e,** Peptide 20–GIPR–G$_s$ complex: left, representative cryo-EM micrograph (scale bar: 40 nm) and two-dimensional class averages

(scale bar: 5 nm); middle, flow chart of cryo-EM data processing; right, local resolution distribution map of the complex and FSC curves of overall refined receptor. The experiments were carried out twice independently with similar results. **f,** Peptide 20–GLP-1R–G_s complex: left, representative cryo-EM micrograph (scale bar: 40 nm) and two-dimensional class averages (scale bar: 5 nm); middle, flow chart of cryo-EM data processing; right, local resolution distribution map of the complex and FSC curves of overall refined receptor. The experiments were repeated independently twice with similar results. **g,** Peptide 20–GCGR–G_s complex: left, representative cryo-EM micrograph (scale bar: 40 nm) and two-dimensional class averages (scale bar: 5 nm); middle, flow chart of cryo-EM data processing; right, local resolution distribution map of the complex and FSC curves of overall refined receptor. The experiments (**a**-**g**) were executed twice independently with similar results.

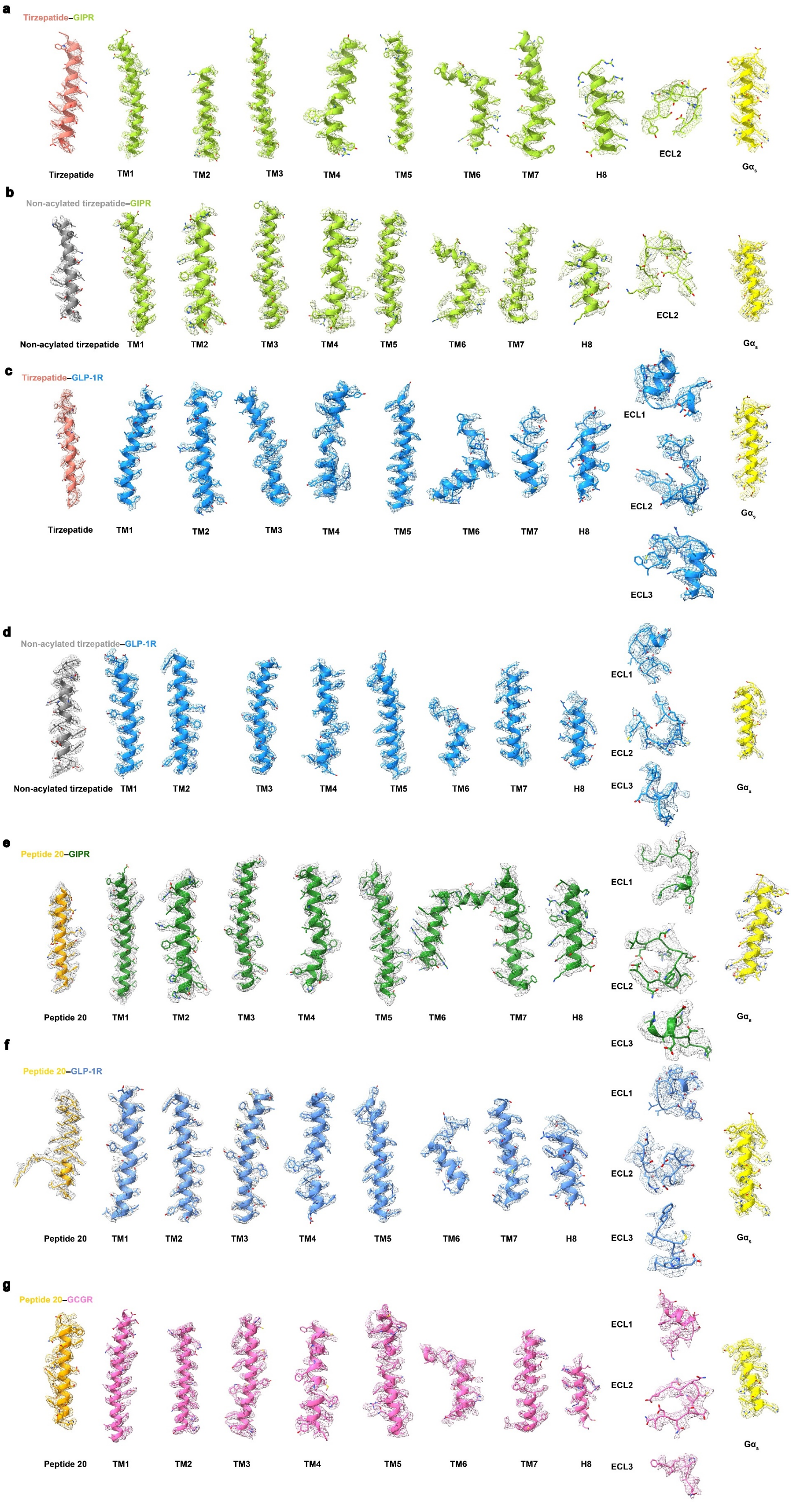

Supplementary Fig. 6 | Near-atomic resolution model of the complexes in the cryo-EM density maps. a, EM density map and model of the tirzepatide–GIPR–G_s complex are shown for all seven-transmembrane α-helices (7TMs), helix 8 and extracellular loop 2 (ECL2) of GIPR, tirzepatide and the α5-helix of the $G\alpha_s$ Ras-like domain. **b,** EM density map and model of the non-acylated tirzepatide–GIPR–G_s complex are shown for 7TMs, helix 8 and ECL2 of GIPR, tirzepatide and the α5-helix of the $G\alpha_s$ Ras-like domain. **c,** EM density map and model of the tirzepatide–GLP-1R–G_s complex are shown for 7TMs, helix 8 and all extracellular loops of GLP-1R, tirzepatide and the α5-helix of the $G\alpha_s$ Ras-like domain. **d,** EM density map and model of the non-acylated tirzepatide–GLP-1R–G_s complex are shown for 7TMs, helix 8 and all

extracellular loops of GLP-1R, tirzepatide and the α5-helix of the $G\alpha_s$ Ras-like domain. **e,** EM density map and model of the peptide 20–GIPR–G_s complex are shown for 7TMs, helix 8 and all extracellular loops of GIPR, peptide 20 and the α5-helix of the $G\alpha_s$ Ras-like domain. **f,** EM density map and model of the peptide 20–GLP-1R–G_s complex are shown for 7TMs, helix 8 and all extracellular loops of GLP-1R, peptide 20 and the α5-helix of the $G\alpha_s$ Ras-like domain. **g,** EM density map and model of the peptide 20–GCGR–G_s complex are shown for 7TMs, helix 8 and all extracellular loops of GCGR, peptide 20 and the α5-helix of the $G\alpha_s$ Ras-like domain.

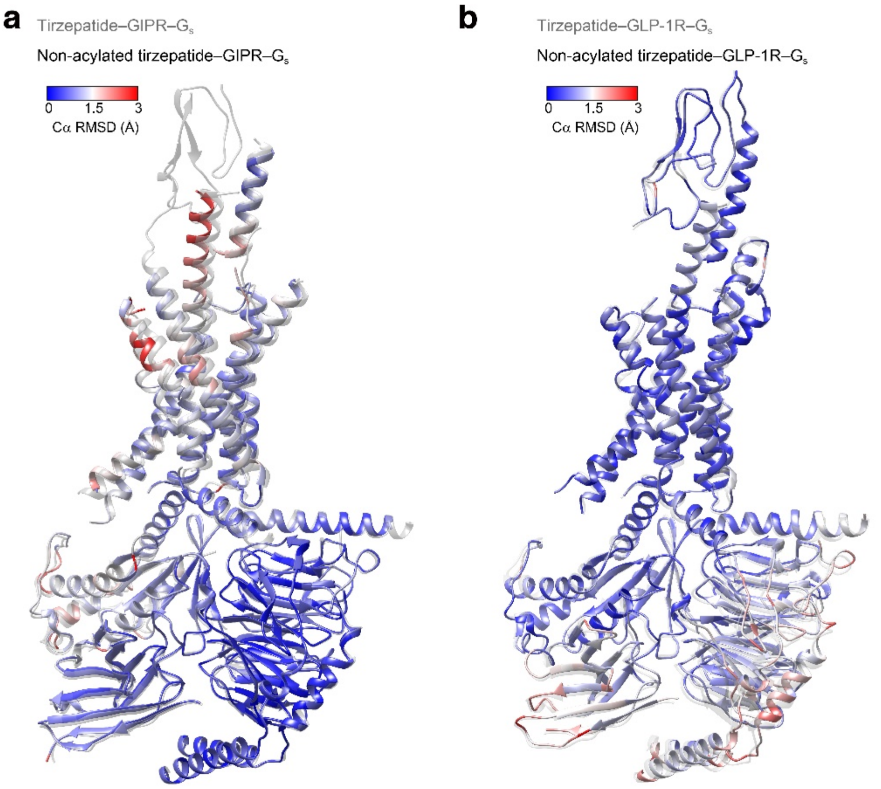

c

Regions	Tirzepatide-bound *vs* non-acylated tirzepatide-bound GIPR			Tirzepatide-bound *vs* non-acylated tirzepatide-bound GLP-1R		
	Residues	Cα (Å)	Heavy atoms (Å)	Residues	Cα (Å)	Heavy atoms (Å)
ECD	1-126			1-137	0.639	1.219
TM1	127-162	1.451	1.770	138-169	0.642	1.181
TM2	167-194	1.081	1.409	175-206	0.575	1.048
TM3	214-247	0.842	0.882	224-255	0.337	0.690
TM4	254-282	0.713	1.409	262-292	0.531	0.962
TM5	296-327	1.008	1.316	302-339	0.360	0.869
TM6	334-362	1.465	1.790	341-371	0.435	1.077
TM7	367-395	1.719	2.000	377-405	0.436	0.929
H8	396-421	0.742	2.135	406-423	0.648	1.170
ECL1	195-213	1.479	2.230	207-223	0.762	1.520
ECL2	283-295	1.233	1.638	293-301	0.629	1.095
ECL3	363-366	3.696	7.200	372-376	0.635	1.444
ICL1	163-166	0.692	1.373	170-174	0.792	0.792
ICL2	248-253	1.974	3.731	256-261	0.524	1.097
Peptide		1.923	2.273		0.786	1.297

Supplementary Fig. 7 | Structural comparison of tirzepatide and non-acylated tirzepatide bound GIPR and GLP-1R. a, Superimposition of the active state GIPR bound by tirzepatide (gray) and non-acylated tirzepatide (colored by Cα RMSD) reveals a high structural similarity with a Cα RMSD of 0.6 Å. **b,** Superimposition of the active state GLP-1R bound by tirzepatide (gray) and non-acylated tirzepatide (colored by Cα RMSD) reveals a high structural similarity with a Cα RMSD of 0.7 Å. **c,** RMSD calculation for different regions of GIPR and GLP-1R. After superimposition of the tirzepatide- and non-acylated tirzepatide-bound GLP-1R or GIPR using the Cα positions of the receptor, RMSD calculation were performed for each region without any fitting using Chimera v1.15.

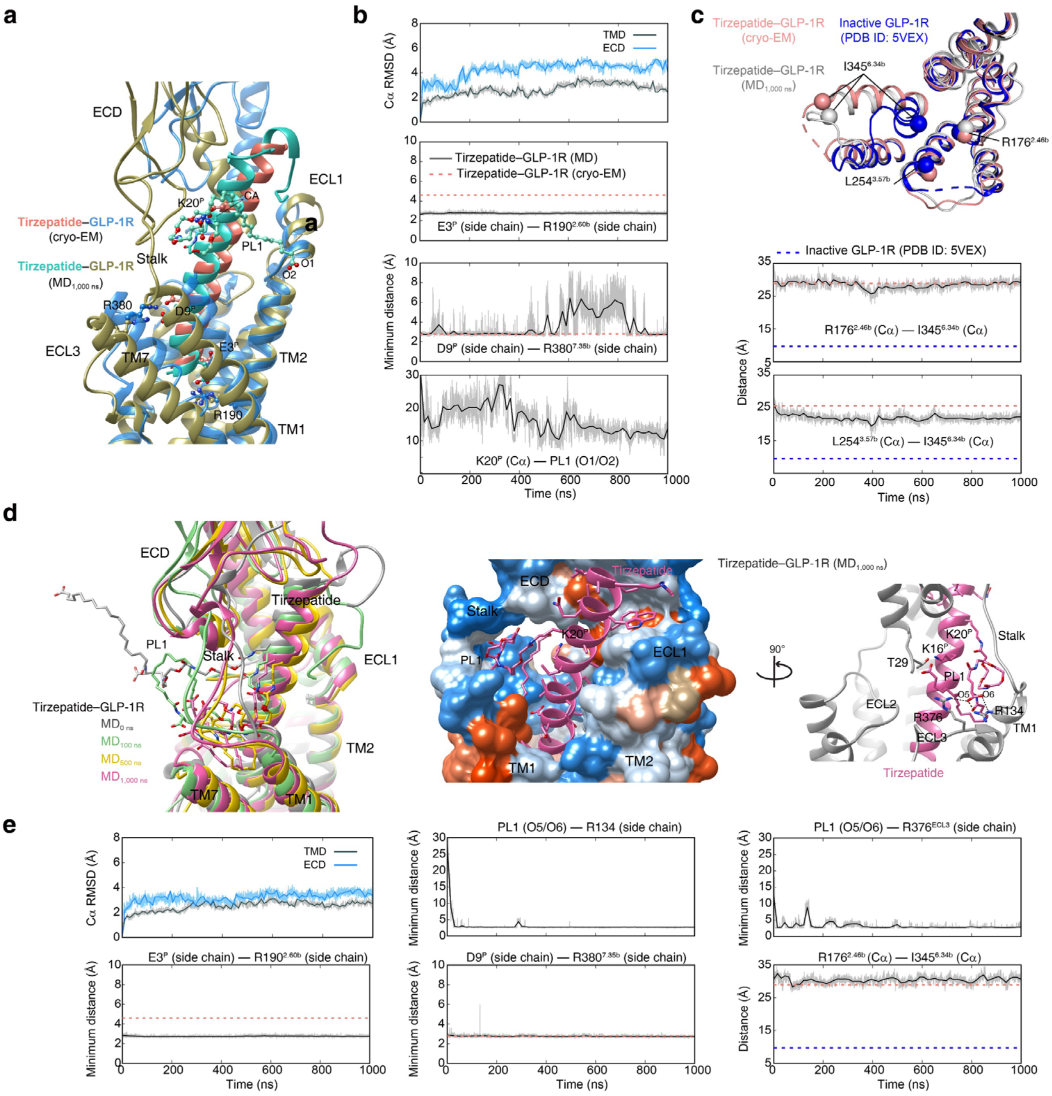

Supplementary Fig. 8 | Molecular dynamics (MD) simulation of GLP-1R bound by tirzepatide. a, Comparison of tirzepatide conformations between simulation snapshot and the cryo-EM structure. The acylated K20^{P} by a γGlu-2×OEG linker and C18 fatty diacid moiety (named as PL1) is shown in sticks. The position of tirzepatide for MD simulation was derived from the cryo-EM structure of tirzepatide–GLP-1R–G$_{s}$ complex, where PL1 initially located over the ECD-TM1 stalk. **b,** Analysis of the MD simulation trajectories in (**a**): top, root mean square deviation (RMSD) of Cα positions of the GLP-1R ECD and TMD, where all snapshots were superimposed on the cryo-EM structure of both tirzepatide- and G$_{s}$-bound GLP-1R ECD and TMD using the Cα atoms, respectively; middle, two representative minimum distances between the side chains of peptide and receptor (E3^{P}–R190$^{2.60b}$ and D9^{P}–R380$^{7.35b}$); bottom, representative minimum distance between peptide and receptor indicates dynamic conformations of the tail of PL1. The thick and thin traces represent moving averages and original, unsmoothed values, respectively. **c,** Movements of the intracellular tip of TM6 during MD simulation. Two representative minimum Cα distance between the intracellular tips of TM6 and TM2 (R176$^{2.46b}$ Cα–I345$^{6.34b}$ Cα) or TM3 (L254$^{3.57b}$ Cα–I345$^{6.34b}$ Cα), indicating that the outward movement of the intracellular part of TM6. The thick and thin traces represent moving averages and original, unsmoothed values, respectively. **d,** Movements of PL1 during MD simulation, whose initial position was manually placed under the ECD-TM1 stalk. Left, comparison of tirzepatide conformation between simulation snapshots and the initial pose; middle, surface representation of the tirzepatide-binding pocket for the finial MD snapshot at 1,000 ns; right, molecular interactions between PL1 and the surrounding residues for the finial MD snapshot at 1,000 ns. **e,** Analysis of the MD simulation trajectories in (**d**): top left, RMSD of Cα positions of the GLP-1R ECD and TMD, where all snapshots were superimposed on the cryo-EM structure of both tirzepatide- and G$_{s}$-bound GLP-1R ECD and TMD using the Cα atoms,

respectively; top middle, minimum distance between the O5/O6 atoms of PL1 and the side chains of R134 during MD simulation; top right, minimum distance between the O5/O6 atoms of PL1 and the side chains of $R376^{ECL3}$ during MD simulation; bottom left, minimum distance between the side chains of $E3^{P}$ and $R190^{2.60b}$; bottom middle, minimum distance between the side chains of $E3^{P}$ and $R380^{7.35b}$; bottom right, minimum Cα distance between the intracellular tips of TM6 and TM2 ($R176^{2.46b}$ Cα–$I345^{6.34b}$ Cα). The thick and thin traces represent moving averages and original, unsmoothed values, respectively.

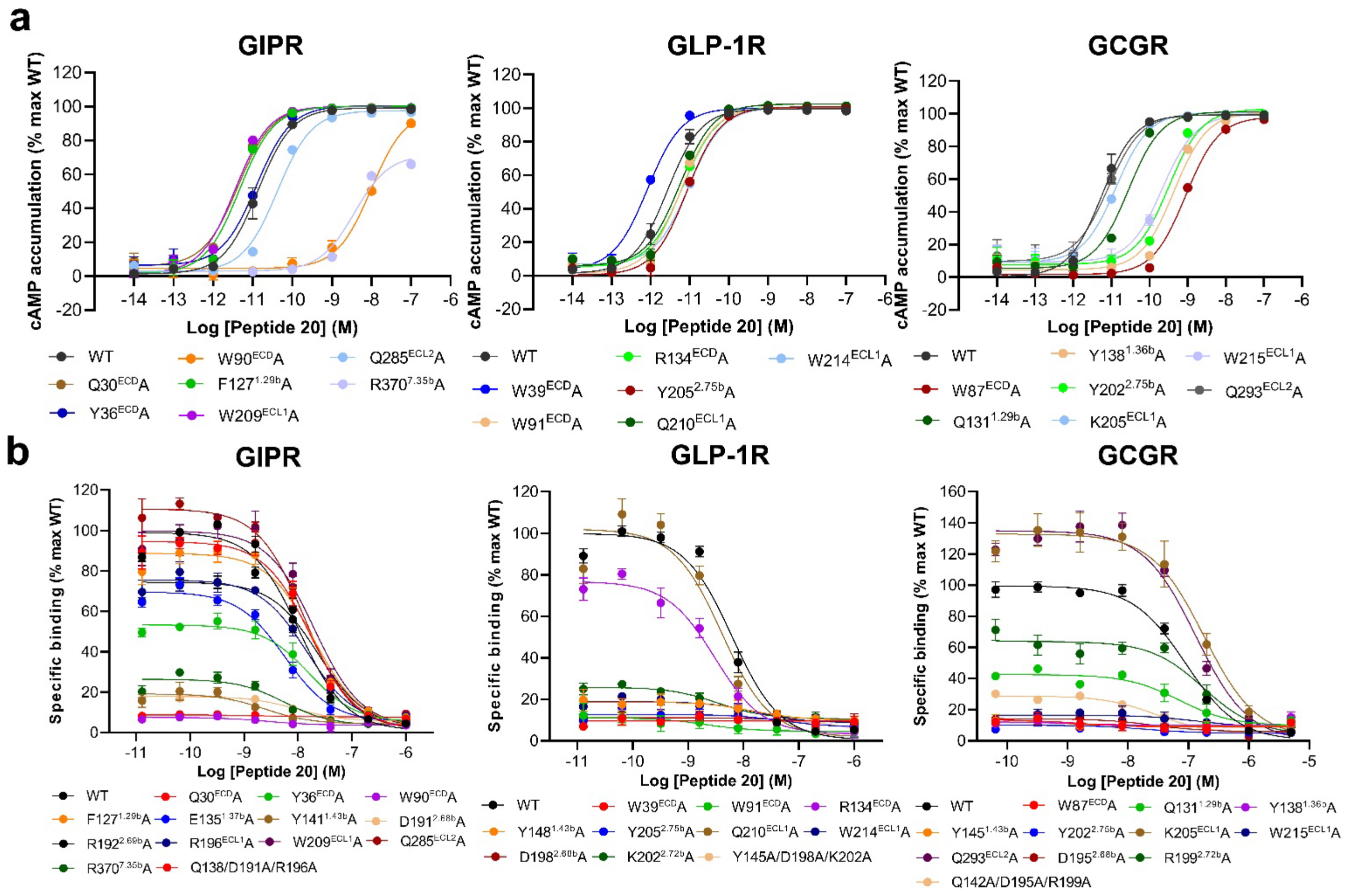

Supplementary Fig. 9 | Effect of receptor mutation on peptide 20-induced cAMP accumulation and receptor binding affinity. a, Signaling profiles of GIPR (left), GLP-1R (middle) and GCGR (right) mutants. cAMP accumulation was measured in wild-type (WT) and single-point mutated GIPR, GLP-1R or GCGR expressing in HEK293T cells, respectively. cAMP accumulation was normalized to the maximum response of the WT and dose-response curves were analyzed using a three-parameter logistic equation. Data were generated and graphed as means ± S.E.M. of at least three independent experiments (*n* = 3-9) performed in quadruplicate. **b,** Binding of peptide 20 to the GIPR (left), GLP-1R (mid) and GCGR (right) mutants in CHO-K1 cells in competition with $^{[125I]}$-GIP$_{1\text{-}42}$, ^{125}I-GLP-1$_{(7\text{-}36)}$NH$_2$ or ^{125}I-GCG. Binding data were analyzed using a three-parameter logistic equation to determine pIC$_{50}$ and span values. Data were generated and graphed as means ± S.E.M. of at least three independent experiments (*n* = 3-10) performed in duplicate. Source data are provided as a Source Data file.

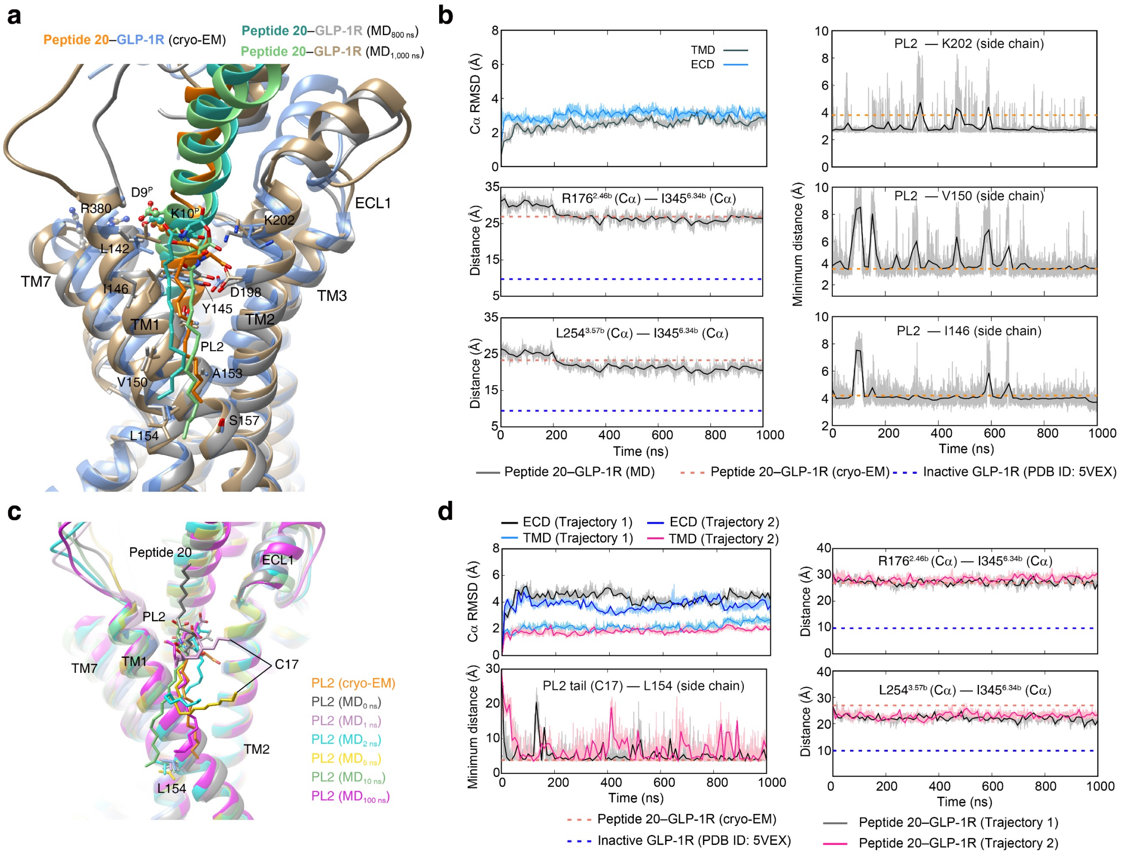

Supplementary Fig. 10 | Molecular dynamics (MD) simulation of GLP-1R bound by peptide 20. a, Comparison of peptide 20 conformations between simulation snapshots and the cryo-EM structure. The lipidated $K10^P$ by a 16-carbon acyl chain (palmitoyl; 16:0) via a γE spacer (named as PL2), with interacting residues shown in sticks. The initial position of peptide 20 for MD simulation was derived from the cryo-EM structure of the peptide 20–GLP-1R–G_s complex, where the palmitate was located in the POPC membrane. **b,** Analysis of the MD simulation trajectories in (**a**): top left, root mean square deviation (RMSD) of Cα positions of the GLP-1R ECD and TMD, where all snapshots were superimposed on the cryo-EM structure of both peptide 20- and G_s-bound GLP-1R ECD and TMD using the Cα atoms, respectively; middle left, representative Cα distance between the intracellular tips of TM2 and TM6 ($R176^{2.46b}$ Cα–$I345^{6.34b}$ Cα), indicating that the outward movement of the intracellular part of TM6; bottom left, representative Cα distance between the intracellular tips of TM3 and TM6 ($L254^{3.57b}$ Cα–$I345^{6.34b}$ Cα), indicating that the outward movement of the intracellular part of TM6; right, representative minimum distance between heavy atoms of PL2 and its interacting residues suggest that PL2 steadily interacts with the TM1-TM2 crevice residues. The thick and thin traces represent moving averages and original, unsmoothed values, respectively. **c,** Movements of the palmitate during MD simulation. The initial position of the palmitate in peptide 20 for MD simulation was manually placed out of the membrane (dark gray). **d,** Analysis of the MD simulation trajectories in (**c**): top left, RMSD of Cα positions of the GLP-1R ECD and TMD, where all snapshots were superimposed on the cryo-EM structure of both peptide 20- and G_s-bound GLP-1R ECD and TMD using the Cα atoms, respectively; bottom left, minimum distance between the PL2 terminal carbon atom (C17) and the side chain of $L154^{1.49b}$, suggesting that PL2 quickly dropped down towards the membrane and finally buried by the lipid environment in a similar pose as seen in the cryo-EM structure. The thick and thin traces represent moving averages and original, unsmoothed values, respectively.

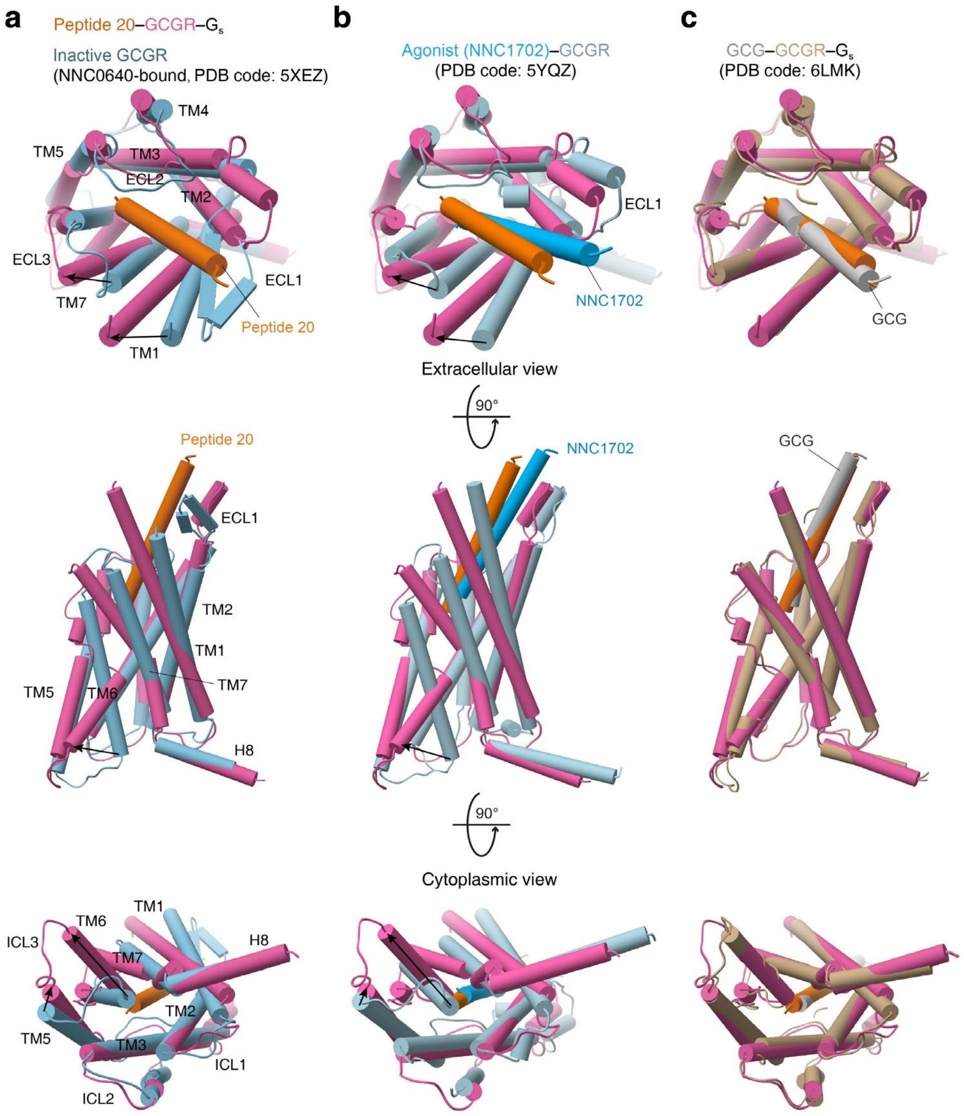

Supplementary Fig. 11 | Conformational changes upon GCGR activation. a-c, Comparison of peptide 20-bound GCGR with inactive (**a**), agonist-bound (**b**) and both GCG-bound and G protein-coupled active GCGR (**c**). G proteins and receptor ECD are omitted for clarity.

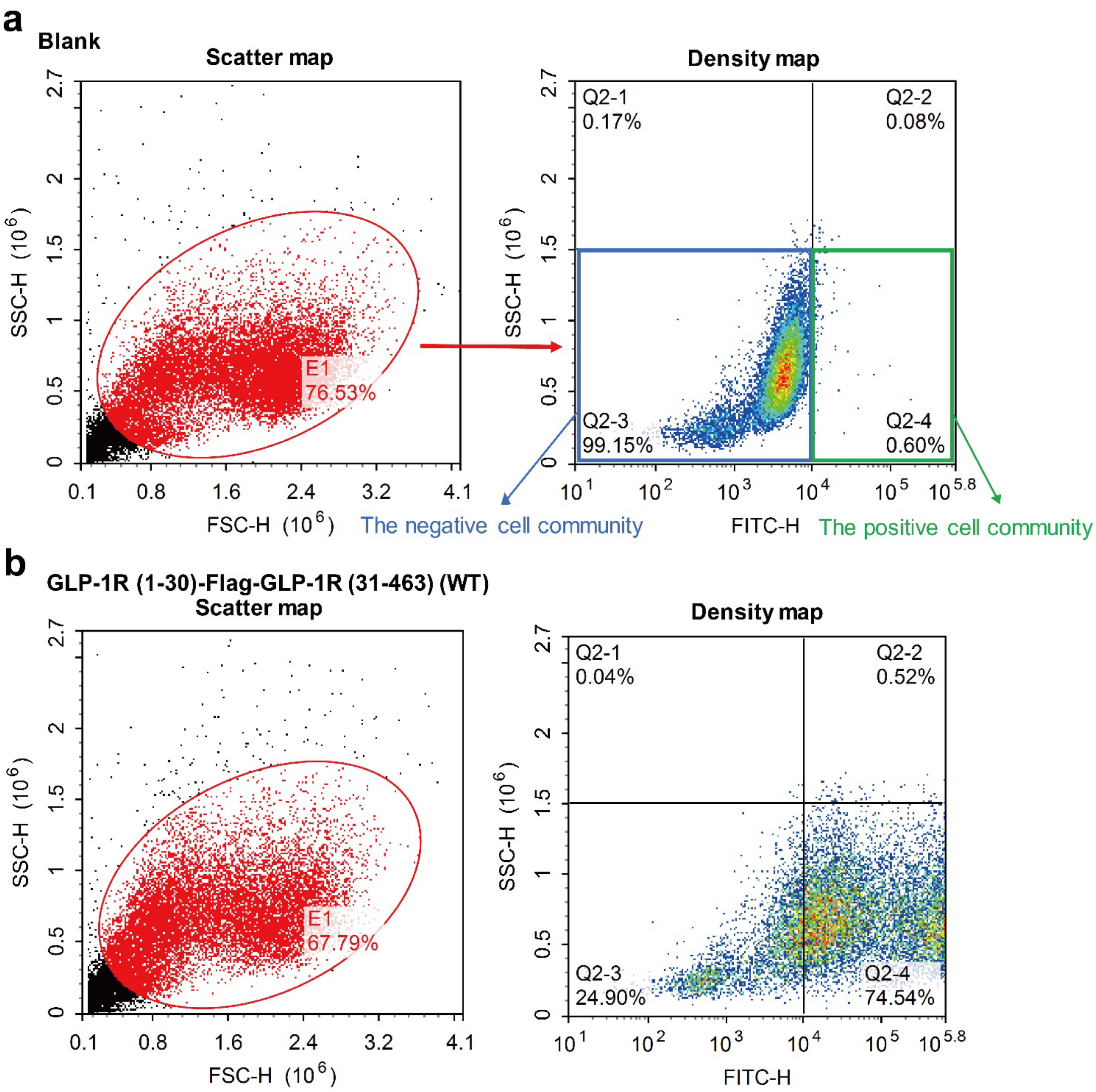

Supplementary Fig. 12 | Gating strategy of the cell surface expression assay. Circle **a** gate E1 in the scatter map (red circle) and the cells shown in the density map are all the cells in the gate E1 of the scatter map. Fluorescence signal intensity (FITC) is presented by density map. With the blank sample (no receptor transfection) as the reference value of background fluorescence signal (**a**), the "quadrant gate" divides the fluorescence signal density map into four quadrants. The third quadrant represents the negative cell community, while the fourth quadrant represents the positive cell community. The expression value of wild-type (WT) receptor (**b**) can be calculated as follows: (M(Q2-4)-M(Q2-3)) × (Q2-4% Parent). The calculation of other mutants is the same as that of the WT receptor, and then normalize with the WT receptor to calculate the relative expression value of the mutants.

Supplementary Table 1 | Mono-, dual and triple agonists at GLP-1R, GCGR or GIPR that entered into clinical development

Drug	Dose form	Manufacturer	NDA/IND	Status	NCT number*	HAb1c (%)	Fasting plasma glucose (mg/dL)	Body weight (kg or %)	Indication
**GLP-1R mono-agonist**									
Exenatide	S.C., b.i.d.	AstraZeneca	2005/FDA	Approved	NCT02533453	-1.21	-34.20	-0.65	T2DM
Lixisenatide	S.C., q.d.	Sanofi-Aventis	2016/FDA	Approved	NCT01973231	-1.24	-29.60	-3.69	T2DM
Liraglutide	S.C., q.d.	Novo Nordisk	2010/FDA	Approved	NCT01117350	-1.81	-36.90 ~ -39.10	-2.99	T2DM
Semaglutide	S.C., q.w.; PO, q.d.	Novo Nordisk	2017/FDA	Approved	NCT03191396	-1.70	-47.70	-5.80	T2DM
Dulaglutide	S.C., q.w.	Eli Lilly	2014/FDA	Approved	NCT02750410	-1.45	-34.20	-0.20	T2DM
Albiglutide	S.C., q.w.	GSK	2014/FDA	Approved	NCT01733758	-1.30	-30.00	-0.04	T2DM
**GLP-1R/GCGR dual agonist**									
JNJ-64565111/ HM12525A/ Efinopegdutide/ MK6024	S.C., q.w.	Merck	2021	Phase 2a	NCT04944992	Unknown	Unknown	Unknown	Obesity, T2DM, NAFLD, NASH
JNJ-54728518	S.C.	Janssen Pharmaceutical	Unknown	Phase 1	Unknown	Unknown	Unknown	Unknown	Obesity
MEDI0382	S.C., q.d.	MedImmune	2017-2019	Phase 2b	NCT03244800	-0.53 ~ -0.82	-27.99 ~ -42.75	-2.41 ~ -4.77	T2DM, NASH, obesity, DKD
MK8521	S.C., q.d.	Merck	2015-2017	Phase 2	NCT02492763	-0.69 ~ -1.41	-19.90 ~ -49.30	-2.10 ~ -4.00	T2DM, hypertension
NN9277/ NNC9204-1177	S.C., q.w.	Novo Nordisk	2017-2020	Phase 1 (stopped)	NCT03308721	Unknown	Unknown	Unknown	Obesity, metabolism and nutrition

									disorder
MOD6031	S.C., q.w.	Prolor Biotech, Opko Health	2016-2016	Phase 1	NCT02692781	Unknown	Unknown	Unknown	Obesity
SAR425899	S.C., q.d.	Sanofi-Aventis	2016-2017	Phase 2 (stopped)	NCT02973321	-1.52 ~ -1.62	-2.32 ~ -2.55	-4.28 ~ -5.33	T2DM, NASH
VPD107/ SP-1373/ ALT-801	S.C., q.w.	Altimmune	2020	Phase 1	NCT04561245	Unknown	Unknown	-6.30	NASH, NAFLD, metastatic melanoma
TT401/ LY2944876/ OPK-88003	S.C., q.w.	Opko	2018-2019	Phase 2 (stopped)	NCT02119819	-0.85 ~ -1.37	-20.88 ~ -31.40	-1.57 ~ -3.41	T2DM
ZP2929	S.C., q.d.	Zealand	2012	Phase 1	Unknown	Unknown	Unknown	Unknown	Obesity, T2DM
BI456906	S.C., q.w.	Zealand, Boehringer Ingelheim	2020	Phase 2	NCT04153929	Unknown	Unknown	Unknown	Obesity, T2DM, NASH
**GIPR/GLP-1R dual agonist**									
LY3298176 (tirzepatide)	S.C., q.w.	Eli Lilly	2018	Phase 3	NCT03311724	-1.70 ~ -2.00	-60.70 ~ -74.20	-5.30 ~ -5.70	T2DM, obesity, NASH
CPD86	S.C., q.d.	Eli Lilly	Unknown	Preclinical	Unknown	Unknown	Unknown	Unknown	Unknown
NN9709/MAR709/ RG7697/ RO6811135	S.C., q.d.	Novo Nordisk/ Marcadia	2014-2015	Phase 2 (stopped)	Unknown	-0.54 ~ -0.77	-15.30 ~ -39.60	-0.90 ~ -3.00	T2DM
SAR438335	Unknown	Sanofi-Aventis	2015-2019	Phase 1 (stopped)	Unknown	Unknown	Unknown	Unknown	T2DM
ZP-DI-70	S.C., q.w.	Zealand	Unknown	Preclinical	Unknown	Unknown	Unknown	Unknown	Unknown
SCO-094	S.C., q.d.	Scohia	2020	Phase 1	Unknown	Unknown	Unknown	Unknown	Obesity, T2DM,

									NASH
**GIPR/GLP-1R/GCGR triple agonist**									
HM15211	S.C., q.w.	Hanmi Pharmaceuticals	2020	Phase 2	NCT04505436	Unknown	Unknown	-13.00 ~ -35.00%	NASH, NAFLD, obesity
MAR423/ NN9423/ NNC9204-1706	S.C., q.d.	Novo Nordisk	2018-2019	Phase 1 (completed)	NCT03661879	Unknown	Unknown	Unknown	Obesity, metabolism and nutrition disorder
SAR441255	S.C.	Sanofi-Aventis	2019	Phase 1 (stopped)	NCT04521738	Unknown	Unknown	Unknown	Overweight
LY3437943	S.C., q.w.	Eli Lilly	2021	Phase 2	Unknown	Unknown	Unknown	-3.50	T2DM, obesity

Data were retrieved from the literature [1-8] and updated from Drugs@FDA, ChemBL and ClinicalTrials.gov databases.

NDA, new drug application; IND, investigational new drug; S.C., subcutaneous injection; PO, *per os* (oral administration); q.d., once daily; b.i.d., twice daily; q.w., once weekly; T2DM, type 2 diabetes mellitus; NASH, non-alcoholic steatohepatitis; NALFD, nonalcoholic fatty liver disease; DKD, diabetic kidney disease.

*U.S. National Library of Medicine number.

Supplementary Table 2 | Cryo-EM data collection, refinement and validation statistics

	**Tirzepatide–GIPR–G$_s$–Nb35 complex**	**Non-acylated tirzepatide–GIPR–G$_s$–Nb35 complex**	**Tirzepatide–GLP-1R–G$_s$–Nb35 complex**	**Non-acylated tirzepatide–GLP-1R–G$_s$–Nb35 complex**	**Peptide 20–GIPR–G$_s$–Nb35 complex**	**Peptide 20–GLP-1R–G$_s$–Nb35 complex**	**Peptide 20–GCGR–G$_s$–Nb35 complex**
**Data collection and processing**							
Magnification	46,685	46,685	46,685	46,685	46,685	46,685	46,685
Voltage (kV)	300	300	300	300	300	300	300
Electron exposure (e^-/Å^2)	80	80	80	80	80	80	80
Defocus range (μm)	-1.2 to -2.2	-1.2 to -2.2	-1.2 to -2.2	-1.2 to -2.2	-1.2 to -2.2	-1.2 to -2.2	-1.2 to -2.2
Pixel size (Å)	1.071	1.071	1.071	1.071	1.071	1.071	1.071
Symmetry imposed	C1	C1	C1	C1	C1	C1	C1
Initial particle images (no.)	4,260,187	7,204,521	4,213,140	5,985,110	5,322,921	4,124,536	3,931,945
Final particle images (no.)	511,557	1,251,553	125,391	452,921	255,256	241,786	383,657
Map resolution (Å)	3.4	3.2	3.4	3.0	3.1	3.0	3.5
FSC threshold	0.143	0.143	0.143	0.143	0.143	0.143	0.143
Map resolution range (Å)	3.1 – 5.4	3.0 – 5.5	3.1 – 6.5	2.7 – 5.0	2.5 – 6.5	2.8 – 4.5	3.1 - 5.4
**Refinement**							
Initial model used (PDB code)	PDB code 7DTY	PDB code 7DTY	PDB code 6X18	PDB code 6X18	PDB code 7DTY	PDB code 6X18	PDB code 6LMK
Model resolution (Å)	3.5	3.3	3.9	3.2	3.5	3.2	3.8
FSC threshold	0.5	0.5	0.5	0.5	0.5	0.5	0.5
Model resolution range (Å)	3.0 – 40	2.9 – 5.0	3.0 – 5.0	3.0 – 5.0	3.0 – 4.0	3.0 – 5.0	2.9 – 5.0
Map sharpening B factor (Å^2)	-168.8	-182.1	-128.0	-148.1	-69.0	-137.2	-191.5
Model composition							
Non-hydrogen atoms	9,368	9,409	9,223	9,223	9,556	9,116	9,040
Protein residues	1,152	1,156	1,158	1,158	1,170	1,141	1,142
Lipids	6	6	0	0	7	0	0
B factors (Å^2)							
Protein	65.7	133.1	172.0	174.0	133.2	159.0	59.5

Ligand	103.8	177.9	0	0	201.6	154.0	74.4
Lipids	101.8	145.8	0	0	148.3	0	0
R.m.s. deviations							
Bond lengths (Å)	0.005	0.005	0.003	0.008	0.005	0.100	0.002
Bond angles (°)	1.014	1.036	0.825	1.021	1.038	1.051	0.552
Validation							
MolProbity score	1.27	1.21	1.46	1.64	1.32	1.78	1.37
Clash score	3.64	4.31	6.96	6.41	4.37	7.61	4.71
Poor rotamers (%)	0	0	0	0	0	0	0
Ramachandran plot							
Favored (%)	97.42	98.15	97.62	95.85	97.48	94.72	97.32
Allowed (%)	2.58	1.85	2.38	4.15	2.52	5.28	2.68
Disallowed (%)	0	0	0	0	0	0	0.00

Supplementary Table 3 | cAMP signaling and receptor binding profiles of tirzepatide, non-acylated tirzepatide and peptide 20 at GIPR or GLP-1R

Receptor	Ligand	cAMP accumulation		Cell surface expression (% WT)	Binding	
		pEC_{50} ± S.E.M.	E_{max} ± S.E.M. (% WT or Tirzepatide)		pIC_{50} ± S.E.M.	Span ± S.E.M. (% WT or Tirzepatide)
GIPR WT	Tirzepatide	10.54 ± 0.06	100.00 ± 1.48	100.00	7.05 ± 0.11	101.56 ± 6.45
	Peptide 20	10.87 ± 0.07	99.18 ± 1.85		7.74 ± 0.10	99.91 ± 4.73
GIPR T345F	Tirzepatide	10.85 ± 0.03**	100.31 ± 0.75	111.73 ± 3.46	6.89 ± 0.16	115.87 ± 11.81
	Peptide 20	11.10 ± 0.08***	99.59 ± 2.09		7.62 ± 0.08	94.89 ± 3.73
GIPR	Tirzepatide	10.54 ± 0.06	100.00 ± 1.48	100.00	6.98 ± 0.08	100.78 ± 4.96
	Non-acylated tirzepatide	11.58 ± 0.06***	99.98 ± 1.50		7.90 ± 0.09***	90.71 ± 3.70
GLP-1R	Tirzepatide	9.43 ± 0.06	100.00 ± 1.73	100.00	7.77 ± 0.18	82.07 ± 6.37
	Non-acylated tirzepatide	11.10 ± 0.03***	100.01 ± 0.88		7.91 ± 0.13	81.36 ± 4.36

cAMP accumulation and binding data were normalized to the maximum response of wild-type (WT) or tirzepatide and dose-response curves were analyzed using a three-parameter logistic equation to obtain pEC_{50} and pIC_{50} values. The experiments were carried out independently at least twice with similar results (n = 3-9). Whole cell binding assay was performed in CHO-K1 cells. Binding data were analyzed using a three-parameter logistic equation to determine pIC_{50} and span values. Data shown are means ± S.E.M. of at least three independent experiments (n = 3-5). Statistically significant differences were determined with a two-tailed Student's t-test. *P< 0.05, **P< 0.01, ***P< 0.001. WT, wild-type.

Supplementary Table 4 | Interaction between tirzepatide and GIPR or GLP-1R

Tirzepatide	GIPR	GLP-1R
Y1^{P}	Hydrogen bond with Q224$^{3.37b}$<br>Hydrophobic contacts with V227$^{3.40b}$ and W296$^{5.36b}$	Hydrogen bond with Q234$^{3.37b}$<br>Hydrophobic contacts with V237$^{3.40b}$ and W306$^{5.36b}$
Aib2^{P}	Hydrophobic contacts with L374$^{7.39b}$ and I378$^{7.43b}$	Hydrophobic contacts with L388$^{7.43b}$
E3^{P}	Hydrogen bond with Y145$^{1.47b}$<br>Salt bridge with R183$^{2.60b}$	Hydrogen bond with Y152$^{1.47b}$<br>Salt bridge with R190$^{2.60b}$
G4^{P}		
T5^{P}	Hydrogen bond with R300$^{5.40b}$	
F6^{P}	Stacking with Y141$^{1.43b}$<br>Hydrophobic contacts with L134$^{1.36b}$, L137$^{1.39b}$, L374$^{7.39b}$ and L378$^{7.43b}$	Stacking with Y148$^{1.43b}$<br>Hydrophobic contacts with L141$^{1.36b}$, L144$^{1.39b}$ and L388$^{7.43b}$
T7^{P}	Hydrogen bond with R190$^{2.67b}$	Hydrogen bond with K197$^{2.67b}$<br>Hydrophobic contacts with T298$^{45.52b}$
S8^{P}	Hydrogen bond with N290^{ECL2}	Hydrogen bond with N300^{ECL2}
D9^{P}	Salt bridge with R370$^{7.35b}$	Salt bridge with R380$^{7.35b}$
Y10^{P}	Hydrogen bond with Q138$^{1.40b}$ and R196^{ECL1}<br>Hydrophobic contacts with L134$^{1.36b}$	Stacking with Y145$^{1.40b}$<br>Hydrophobic contacts with L141$^{1.36b}$ and L201$^{2.71b}$
S11^{P}	Hydrogen bond with E288$^{45.52b}$	Hydrogen bond with Y205^{ECL1} and T298$^{45.52b}$<br>Hydrophobic contacts with L201$^{2.71b}$
I12^{P}		
Aib13^{P}	Hydrophobic contacts with R131$^{1.33b}$	Hydrophobic contacts with L141$^{1.36b}$
L14^{P}	Hydrophobic contacts with R196^{ECL1} and P197^{ECL1}	Hydrophobic contacts with Y205^{ECL1}
D15^{P}	Salt bridge with R289^{ECL2}	Hydrogen bond with S31^{ECD}, L32^{ECD}, Y205^{ECL1}<br>Salt bridge with R299^{ECL2}
K16^{P}	Stacking with F127$^{1.29b}$	
I17^{P}	Hydrophobic contacts with F127$^{1.29b}$, L128$^{1.30b}$ and R131$^{1.33b}$	
A18^{P}	Hydrophobic contacts with P197^{ECL1}	Hydrophobic contacts with L32^{ECD}
Q19^{P}	Hydrogen bond with Q30^{ECD}	Hydrogen bond with S31^{ECD}
K20^{P}	Hydrogen bond with N124^{ECD}	Salt bridge with E128^{ECD}
A21^{P}		
F22^{P}	Stacking with Y36^{ECD} and W39^{ECD}<br>Hydrophobic contacts with L35^{ECD}	Stacking with W39^{ECD} and W214^{ECL1}<br>Hydrophobic contacts with V36^{ECD}
V23^{P}	Hydrophobic contacts with W91^{ECD}	Hydrophobic contacts with P90^{ECD} and W91^{ECD}
Q24^{P}		
W25^{P}	Stacking with W39^{ECD} and Y200^{ECL1}	Stacking with W39^{ECD} and W214^{ECL1}
L26^{P}	Hydrophobic contacts with W39^{ECD}	Hydrophobic contacts with W39^{ECD} and Y88^{ECD}
I27^{P}	Hydrogen bond with R113^{ECD}<br>Hydrophobic contacts with Y68^{ECD}	Hydrogen bond with R121^{ECD}<br>Hydrophobic contacts with Y69^{ECD}
A28^{P}		

Supplementary Table 5 | Interaction between peptide 20, GIPR, GLP-1R and GCGR

Peptide 20	GIPR	GLP-1R	GCGR
H1^{P}	Hydrogen bond with R183$^{2.60b}$	Hydrogen bond with Q234$^{3.37b}$<br>Hydrophobic contacts with V237$^{3.40b}$ and W306$^{5.36b}$	Hydrogen bond with Q232$^{3.37b}$<br>Hydrophobic contacts with L235$^{3.40b}$ and W304$^{5.36b}$
Aib2^{P}	Hydrophobic contacts with L374$^{7.39b}$ and I378$^{7.43b}$	Hydrophobic contacts with L388$^{7.43b}$	Hydrophobic contacts with L386$^{7.43b}$
Q3^{P}	Hydrogen bond with Y145$^{1.47b}$	Hydrogen bond with K197$^{2.67b}$	Hydrogen bond with Y149$^{1.47b}$
G4^{P}		Hydrogen bond with N300^{ECL2}	
T5^{P}		Hydrogen bond with D372^{ECL3}	
F6^{P}	Stacking with Y141$^{1.43b}$<br>Hydrophobic contacts with L374$^{7.39b}$ and L378$^{7.43b}$	Stacking with Y148$^{1.43b}$<br>Hydrophobic contacts with L141$^{1.36b}$, L144$^{1.39b}$ and L388$^{7.43b}$	Stacking with Y138$^{1.36b}$ and Y145$^{1.43b}$<br>Hydrophobic contacts with Q142$^{1.40b}$ and L386$^{7.43b}$
T7^{P}	Hydrogen bond with R190$^{2.67b}$	Hydrogen bond with K197$^{2.67b}$	Hydrogen bond with T296$^{45.52b}$
S8^{P}	Hydrogen bond with N290^{ECL2}	Hydrogen bond with N300^{ECL2}	Hydrogen bond with N298^{ECL2}
D9^{P}	Salt bridge with R370$^{7.35b}$	Salt bridge with R380$^{7.35b}$	Hydrogen bond with R378$^{7.35b}$
K10^{P}	Hydrogen bond with Q138$^{1.40b}$<br>Hydrophobic contacts with L134$^{1.36b}$	Stacking with Y145$^{1.40b}$<br>Hydrophobic contacts with L141$^{1.36b}$	Hydrogen bond with S139$^{1.37b}$, Q142$^{1.40b}$ and R199$^{2.72b}$<br>Hydrophobic contacts with Y138$^{1.36b}$
S11^{P}	Hydrogen bond with E288$^{45.52b}$	Hydrogen bond with Y205^{ECL1} and T298$^{45.52b}$	Hydrogen bond with S297^{ECL2}
K12^{P}			
Y13^{P}	Hydrogen bond with F127$^{1.29b}$<br>Hydrophobic contacts with R131$^{1.33b}$ and L134$^{1.36b}$		Hydrogen bond with Q131$^{1.29b}$
L14^{P}	Hydrophobic contacts with P197^{ECL1}	Hydrophobic contacts with Y205^{ECL1}	Hydrophobic contacts with L198$^{2.71b}$ and Y202$^{2.75b}$
D15^{P}	Salt bridge with R289^{ECL2}<br>Hydrogen bond with Q30^{ECD} and A32^{ECD}	Salt bridge with R299^{ECL2}<br>Hydrogen bond with S31^{ECD} and L32^{ECD}	Hydrogen bond with Y202$^{2.75b}$ and V28^{ECD}
E16^{P}			
R17^{P}			Hydrogen bond with Y202$^{2.75b}$
A18^{P}	Hydrophobic contacts with P199^{ECL1}	Hydrophobic contacts with L32^{ECD}	Hydrophobic contacts with Y202$^{2.75b}$
A19^{P}	Hydrophobic contacts with L35^{ECD}	Hydrophobic contacts with L32^{ECD}	Hydrophobic contacts with L32^{ECD}
Q20^{P}	Hydrogen bond with N120^{ECD}		
D21^{P}		Hydrogen bond with Q210^{ECL1}	Hydrogen bond with I206^{ECL1}
F22^{P}	Stacking with Y36^{ECD} and W39^{ECD}<br>Hydrophobic contacts with L35^{ECD}	Stacking with W39^{ECD} and W214^{ECL1}<br>Hydrophobic contacts with V36^{ECD}	Stacking with W36^{ECD}<br>Hydrophobic contacts with L32^{ECD}
V23^{P}	Hydrophobic contacts with L88^{ECD}	Hydrophobic contacts with L89^{ECD}	Hydrophobic contacts with L85^{ECD}
Q24^{P}			
W25^{P}	Stacking with W39^{ECD}	Stacking with W39^{ECD} and W214^{ECL1}	Hydrophobic contacts with I206^{ECL1}
L26^{P}	Hydrophobic contacts with W39^{ECD} and M67^{ECD}	Hydrophobic contacts with W39^{ECD}	Hydrophobic contacts with W36^{ECD} and Y84^{ECD}
L27^{P}	Hydrogen bond with R113^{ECD}<br>Hydrophobic contacts with Y68^{ECD}	Hydrogen bond with R121^{ECD}<br>Hydrophobic contacts with Y69^{ECD}	Hydrophobic contacts with Y65^{ECD}
D28^{P}			Hydrogen bond with P114^{ECL1}

Supplementary Table 6 | cAMP signaling profiles of endogenous agonists, multi-targeting agonists and approved GLP-1 analogs at GIPR, GLP-1R and GCGR

Peptide	GIPR		GLP-1R		GCGR	
	pEC_{50} ± S.E.M.	E_{max} ± S.E.M. (% max GIP_{1-42})	pEC_{50} ± S.E.M.	E_{max} ± S.E.M. (% max GLP-$1_{(7-36)}NH_2$)	pEC_{50} ± S.E.M.	E_{max} ± S.E.M. (% max GCG)
GIP_{1-42}	11.81 ± 0.06	100.25 ± 1.55	N.A.	N.A.	N.A.	N.A.
GLP-$1_{(7-36)}NH_2$	N.A.	N.A.	11.58 ± 0.06	100.62 ± 1.72	N.A.	N.A.
GCG	N.A.	N.A.	9.37 ± 0.07***	103.25 ± 3.50	11.33 ± 0.06	100.33 ± 1.66
Semaglutide	N.A.	N.A.	11.24 ± 0.07**	99.40 ± 2.12	N.A.	N.A.
Tirzepatide	10.90 ± 0.05***	100.30 ± 1.73	9.97 ± 0.05***	100.79 ± 2.08	N.A.	N.A.
Peptide 20	11.35 ± 0.05***	100.26 ± 1.48	11.91 ± 0.04**	99.58 ± 1.17	11.58 ± 0.06*	101.20 ± 1.68
Albiglutide	N.A.	N.A.	11.02 ± 0.05***	100.13 ± 1.66	N.A.	N.A.
Liraglutide	N.A.	N.A.	11.27 ± 0.05**	99.57 ± 1.62	N.A.	N.A.
Lixisenatide	N.A.	N.A.	10.02 ± 0.07***	100.51 ± 2.68	N.A.	N.A.

cAMP accumulation assay was performed in HEK293T cells and the data were analyzed using a three-parameter logistic equation to determine pEC_{50} and E_{max} values. E_{max} values are expressed as a percentage of the GIP_{1-42}, GLP-$1_{(7-36)}NH_2$ or GCG, related to GIPR, GLP-1R or GCGR. Data shown are means ± S.E.M. of four independent experiments (n = 4). To determine statistical difference, one-way ANOVA were used in GIPR and GLP-1R, and two-tailed Student's t-test was used in GCGR (*P< 0.05, **P< 0.01, ***P< 0.001). N.A., not active.

Supplementary Table 7 | Effects of ligand-binding pocket residue mutation on tirzepatide-induced cAMP responses and receptor binding profiles

Receptor	Mutant	cAMP accumulation		Receptor binding	
		pEC_{50} ± S.E.M.	E_{max} ± S.E.M. (% WT)	pIC_{50} ± S.E.M.	Span ± S.E.M. (% WT)
GIPR	WT	10.53 ± 0.06	100.00 ± 1.48	7.01 ± 0.11	102.92 ± 6.94
	$Y36^{ECD}A$	10.23 ± 0.05**	99.70 ± 1.46	6.56 ± 0.42	40.56 ± 12.64***
	$W90^{ECD}A$	8.11 ± 0.05***	100.98 ± 2.13	N.B.	3.97 ± 2.47***
	$Y141^{1.43b}A$	8.54 ± 0.05***	101.13 ± 1.55	N.B.	6.77 ± 1.72***
	$Y145^{1.47b}A$	9.22 ± 0.04***	100.45 ± 1.08	N.B.	8.58 ± 1.56***
	$D191^{2.68b}A$	9.30 ± 0.05***	100.92 ± 1.50	N.B.	5.03 ± 1.41***
	$R370^{7.35b}A$	9.18 ± 0.05***	101.38 ± 1.42	N.B.	10.40 ± 2.67***
GLP-1R	WT	9.44 ± 0.06	100.00 ± 1.73	8.36 ± 0.16	77.63 ± 5.70
	$Y148^{1.43b}A$	8.46 ± 0.07***	101.52 ± 2.33	N.B.	12.31 ± 2.51***
	$Y152^{1.47b}A$	8.46 ± 0.07***	101.49 ± 2.25	N.B.	11.22 ± 2.82***
	$R190^{2.60b}A$	6.52 ± 0.03***	101.79 ± 2.41*	N.B.	6.78 ± 1.41***
	$K197^{2.67b}A$	6.41 ± 0.04***	110.54 ± 2.77	N.B.	5.75 ± 0.91***
	$Y205^{2.75b}A$	7.94 ± 0.03***	100.68 ± 1.35	N.B.	7.94 ± 2.42***
	$W214^{ECL1}A$	8.76 ± 0.03***	99.10 ± 1.05	N.B.	11.88 ± 1.32***
	$R299^{ECL2}A$	8.32 ± 0.06***	100.96 ± 2.36	N.B.	8.59 ± 1.61***
	$N300^{ECL2}A$	6.60 ± 0.04***	101.10 ± 2.77	N.B.	6.23 ± 1.66***

cAMP accumulation assay was performed in HEK293T cells. All the mutant constructs were made by single-point mutation in the setting of the wild-type (WT) construct. cAMP accumulation data were analyzed using a three-parameter logistic equation to determine pEC_{50} and E_{max} values. E_{max} values for mutants are expressed as a percentage of the WT. Whole cell binding assay was performed in CHO-K1 cells. Binding data were analyzed using a three-parameter logistic equation to determine pIC_{50} and span values. Data shown are means ± S.E.M. of at least three independent experiments (*n* = 3-4). One-way ANOVA were used to determine statistical difference (*P< 0.05, **P< 0.01, ***P< 0.001). N.B., no binding was detected.

Supplementary Table 8 | Effects of the ligand-binding pocket residue mutation on peptide 20-induced cAMP signaling and receptor binding profiles at GIPR, GLP-1R and GCGR

Receptor	Mutant	cAMP accumulation		Receptor binding	
		pEC$_{50}$ ± S.E.M.	E$_{max}$ ± S.E.M. (% WT)	pIC$_{50}$ ± S.E.M.	Span ± S.E.M. (% WT)
GIPR	WT	10.87 ± 0.07	99.18 ± 1.85	7.97 ± 0.08	97.43 ± 3.89
	Q30^{ECD}A	10.37 ± 0.06*	100.12 ± 1.79	7.77 ± 0.10	92.75 ± 4.50
	Y36^{ECD}A	10.91 ± 0.06	100.40 ± 1.62	7.79 ± 0.12	48.19 ± 2.73***
	W90^{ECD}A	8.01 ± 0.08***	98.03 ± 4.59	8.57 ± 0.62	3.87 ± 1.12***
	F127$^{1.29b}$A	11.34 ± 0.07*	100.06 ± 1.64	7.70 ± 0.10	85.53 ± 4.36
	E135$^{1.37b}$A	10.37 ± 0.05*	100.18 ± 1.44	8.25 ± 0.08	64.00 ± 2.46***
	Y141$^{1.43b}$A	8.43 ± 0.05***	75.07 ± 1.79***	8.62 ± 0.28	14.88 ± 1.95***
	R196^{ECL1}A	10.33 ± 0.05*	98.53 ± 1.43	7.84 ± 0.10	71.10 ± 3.42***
	W209^{ECL1}A	11.44 ± 0.06**	100.18 ± 1.45	7.70 ± 0.13	96.83 ± 6.13
	Q285^{ECL2}A	10.38 ± 0.05*	97.56 ± 1.37	7.87 ± 0.07	108.98 ± 3.60
	R370$^{7.35b}$A	8.43 ± 0.09***	71.47 ± 2.85***	8.25 ± 0.19	22.84 ± 2.01***
	D191$^{2.68b}$A	8.32 ± 0.30***	51.71 ± 5.96***	8.04 ± 0.18	12.29 ± 1.02***
	R192$^{2.69b}$A	10.52 ± 0.07	101.33 ± 1.91	7.62 ± 0.12	71.60 ± 4.11***
	Q138A/D191A/R196A	7.52 ± 0.35***	56.32 ± 12.18***	8.62 ± 1.96	1.03 ± 0.94***
GLP-1R	WT	11.57 ± 0.06	99.63 ± 1.37	8.21 ± 0.06	99.44 ± 2.88
	W39^{ECD}A	12.08 ± 0.05***	100.00 ± 1.04	N.B.	N.B.
	W91^{ECD}A	11.22 ± 0.05**	100.40 ± 1.33	8.69 ± 0.85	6.51 ± 2.59***
	R134^{ECD}A	11.18 ± 0.04***	100.64 ± 1.08	8.52 ± 0.10	73.44 ± 3.42
	Y148$^{1.43b}$A	11.30 ± 0.05*	101.19 ± 1.26	7.81 ± 0.63	8.73 ± 2.65***
	Y205$^{2.75b}$A	11.07 ± 0.04***	100.75 ± 1.08	7.30 ± 0.99	6.01 ± 3.17***
	Q210^{ECL1}A	11.25 ± 0.05**	102.50 ± 1.27	8.39 ± 0.11	100.10 ± 5.13
	W214^{ECL1}A	11.01 ± 0.04***	100.44 ± 0.94	7.77 ± 0.35	10.25 ± 1.71***
	D198$^{2.68b}$A	11.35 ± 0.07	99.88 ± 1.82	7.28 ± 1.65	1.88 ± 1.66***
	K202$^{2.72b}$A	11.59 ± 0.08	100.46 ± 1.97	8.46 ± 0.26	15.32 ± 1.82***
	Y145A/D198A/K202A	10.41 ± 0.06***	99.91 ± 1.67	5.75 ± 2.27	10.85 ± 36.78***
GCGR	WT	11.25 ± 0.07	99.32 ± 1.73	7.05 ± 0.06	99.29 ± 3.10
	W87^{ECD}A	9.05 ± 0.04***	98.11 ± 1.54	8.82 ± 1.20	4.68 ± 2.95***
	Q131$^{1.29b}$A	10.55 ± 0.06***	101.29 ± 1.59	7.16 ± 0.21	32.87 ± 3.34***
	Y138$^{1.36b}$A	9.35 ± 0.07***	100.93 ± 2.39	8.94 ± 2.20	2.08 ± 2.48***
	Y145$^{1.43b}$A	8.72 ± 0.04***	102.71 ± 1.79	9.34 ± 1.42	4.34 ± 3.92***
	Y202$^{2.75b}$A	9.49 ± 0.07***	102.88 ± 2.48	7.73 ± 0.73	5.12 ± 1.71***
	K205^{ECL1}A	10.88 ± 0.05**	100.79 ± 1.28	6.74 ± 0.13	130.88 ± 9.06***
	W215^{ECL1}A	9.66 ± 0.07***	99.60 ± 2.02	6.84 ± 0.70	7.72 ± 2.74***
	Q293^{ECL2}A	11.08 ± 0.07	99.45 ± 1.63	6.88 ± 0.09	135.17 ± 6.41***
	D195$^{2.68b}$A	9.57 ± 0.06***	102.24 ± 2.10	7.59 ± 0.45	8.34 ± 1.73***
	R199$^{2.72b}$A	10.89 ± 0.05**	99.72 ± 1.47	6.75 ± 0.18	59.87 ± 5.43***
	Q142A/D195A/R199A	9.28 ± 0.06***	100.46 ± 2.37	7.52 ± 0.16	24.37 ± 1.80***

cAMP accumulation assay was performed in HEK293T cells. All the mutant constructs were made by single-point mutation in the setting of the wild-type (WT) construct. cAMP accumulation data were analyzed using a three-parameter logistic equation to determine pEC$_{50}$ and E$_{max}$ values. E$_{max}$ values for mutants are expressed as a percentage of the WT. Whole cell binding assay was performed in CHO-K1 cells. Binding data were analyzed using a three-parameter logistic equation to determine pIC$_{50}$ and span values. Data shown are means ± S.E.M. of at least three independent experiments (n = 3-10). One-way ANOVA were used to determine statistical difference (*$P < 0.05$, **$P < 0.01$, ***$P < 0.001$). N.B., no binding was

detected.

Supplementary Table 9 | Effects of ligand-binding pocket residue mutation on receptor expression

Mutant	Cell surface expression (% WT)	Mutant	Cell surface expression (% WT)	Mutant	Cell surface expression (% WT)
HA-Flag-3GSA-GIPR (22-466) (WT)	100.00	GLP-1R (1-30)-Flag-GLP-1R (31-463) (WT)	100.00	GCGR (1-477)-V5-6×His (WT)	100.00
HA-Flag-3GSA-GIPR (22-466) Q30^{ECD}A	110.40 ± 0.50	GLP-1R (1-30)-Flag-GLP-1R (31-463) W39^{ECD}A	63.94 ± 4.07**	GCGR (1-477) W87^{ECD}A-V5-6×His	95.85 ± 3.41
HA-Flag-3GSA-GIPR (22-466) Y36^{ECD}A	85.32 ± 2.63	GLP-1R (1-30)-Flag-GLP-1R (31-463) W91^{ECD}A	75.09 ± 6.92	GCGR (1-477) Q131$^{1.29b}$A-V5-6×His	84.62 ± 4.14
HA-Flag-3GSA-GIPR (22-466) W90^{ECD}A	69.26 ± 0.76***	GLP-1R (1-30)-Flag-GLP-1R (31-463) R134^{ECD}A	157.30 ± 6.47***	GCGR (1-477) Y138$^{1.36b}$A-V5-6×His	92.99 ± 2.59
HA-Flag-3GSA-GIPR (22-466) F127$^{1.29b}$A	88.98 ± 5.84	GLP-1R (1-30)-Flag-GLP-1R (31-463) Y148$^{1.43b}$A	117.30 ± 5.07	GCGR (1-477) Y145$^{1.43b}$A-V5-6×His	109.40 ± 5.01
HA-Flag-3GSA-GIPR (22-466) E135$^{1.37b}$A	101.60 ± 3.76	GLP-1R (1-30)-Flag-GLP-1R (31-463) Y205$^{2.75b}$A	99.60 ± 4.82	GCGR (1-477) Y202$^{2.75b}$A-V5-6×His	92.68 ± 7.85
HA-Flag-3GSA-GIPR (22-466) Y141$^{1.43b}$A	136.60 ± 3.31***	GLP-1R (1-30)-Flag-GLP-1R (31-463) Q210^{ECL1}A	115.50 ± 5.84	GCGR (1-477) K205^{ECL1}A-V5-6×His	113.40 ± 8.54
HA-Flag-3GSA-GIPR (22-466) Y145$^{1.47b}$A	71.66 ± 2.74**	GLP-1R (1-30)-Flag-GLP-1R (31-463) Y152$^{1.47b}$A	5.94 ± 1.33***	GCGR (1-477) W215^{ECL1}A-V5-6×His	97.69 ± 4.88
HA-Flag-3GSA-GIPR (22-466) R196^{ECL1}A	79.86 ± 2.52*	GLP-1R (1-30)-Flag-GLP-1R (31-463) W214^{ECL1}A	91.22 ± 9.67	GCGR (1-477) Q293^{ECL2}A-V5-6×His	109.10 ± 6.94
HA-Flag-3GSA-GIPR (22-466) W209^{ECL1}A	91.34 ± 1.98	GLP-1R (1-30)-Flag-GLP-1R (31-463) D198$^{2.68b}$A	85.09 ± 16.50	GCGR (1-477) D195$^{2.68}$bA-V5-6×His	61.64 ± 4.18***
HA-Flag-3GSA-GIPR (22-466) Q285^{ECL2}A	95.57 ± 5.06	GLP-1R (1-30)-Flag-GLP-1R (31-463) K202$^{2.72b}$A	43.73 ± 2.13***	GCGR (1-477) R199$^{2.72b}$A-V5-6×His	58.84 ± 3.14***
HA-Flag-3GSA-GIPR (22-466) R370$^{7.35b}$A	129.40 ± 2.66***	GLP-1R (1-30)-Flag-GLP-1R (31-463) R190$^{2.60b}$A	39.29 ± 1.73***	GCGR (1-477) Q142A/D195A/R199A-V5-6×His	40.90 ± 4.93***
HA-Flag-3GSA-GIPR (22-466) D191$^{2.68b}$A	70.48 ± 11.02**	GLP-1R (1-30)-Flag-GLP-1R (31-463) K197$^{2.67b}$A	49.08 ± 2.99***		
HA-Flag-3GSA-GIPR (22-466) R192$^{2.69b}$A	108.00 ± 8.49	GLP-1R (1-30)-Flag-GLP-1R (31-463) R299^{ECL2}A	47.93 ± 4.23***		
HA-Flag-3GSA-GIPR (22-466) Q138A/D191A/R196A	77.43 ± 0.67*	GLP-1R (1-30)-Flag-GLP-1R (31-463) N300^{ECL2}A	80.26 ± 1.88		
		GLP-1R (1-30)-Flag-GLP-1R (31-463) Y145A/D198A/K202A	38.63 ± 4.27***		

Cell surface expression was assessed by FACS. Values were normalized to the wild-type (WT, shown as percentage) in HEK293T cells. All the mutant constructs were modified by single-point mutation in the setting of the WT construct. Data shown are means ± S.E.M. of at least three independent experiments (*n* = 3-6). One-way ANOVA were used to determine statistical difference (*P< 0.05, **P< 0.01, ***P< 0.001).

Supplementary Table 10 | Signaling profiles of mono- and triple agonists at GIPR, GLP-1R and GCGR

Receptor	Agonist	Casein						BSA					
		cAMP accumulation		β-arrestin 2 recruitment		Binding		cAMP accumulation		β-arrestin 2 recruitment		Binding	
		pEC50 ± S.E.M.	Emax ± S.E.M.	pEC50 ± S.E.M.	Emax ± S.E.M.	pIC50 ± S.E.M.	Span ± S.E.M.	pEC50 ± S.E.M	Emax ± S.E.M.	pEC50 ± S.E.M	Emax ± S.E.M.	pIC50 ± S.E.M.	Span ± S.E.M.
GIPR	GIP_{1-42}	12.21 ± 0.04	100.10 ± 1.15	8.01 ± 0.03	100.00 ± 1.10	8.61 ± 0.08	100.00± 3.47	11.81 ± 0.06	100.25 ± 1.55	7.79 ± 0.06	100.00 ± 1.90	8.55 ± 0.31	100.00 ± 13.84
	Peptide 20	10.88 ± 0.06[d]	100.44 ± 1.51	N.A.	N.A.	8.47 ± 0.15	99.92 ± 6.37	11.35 ± 0.05[a]	99.91 ± 1.18	N.A.	N.A.	7.88 ± 0.37	85.89 ± 15.12
	Non-lipidated peptide 20	7.94 ± 0.13[d,h]	88.53 ± 6.52	N.A.	N.A.	6.77 ± 0.32[b,f]	83.09 ± 17.61	8.24 ± 0.13 [d,h]	99.40 ± 6.44	N.A.	N.A.	7.29 ± 0.50	78.13 ± 20.74
GLP-1R	$GLP\text{-}1_{(7\text{-}36)}NH_2$	12.53 ± 0.08	100.00 ± 1.83	7.79 ± 0.02	100.00 ± 0.68	7.88 ± 0.17	100 ± 7.81	11.58 ± 0.06	100.62 ± 1.72	7.49 ± 0.07	100.00 ± 2.61	8.19 ± 0.11	100.00 ± 4.90
	Peptide 20	12.07 ± 0.06[b]	98.50 ± 1.33	7.58 ± 0.05	80.97 ± 1.28[b]	8.74 ± 0.21[a]	85.25 ± 8.33	11.91 ± 0.04[b]	99.24 ± 0.96	7.18 ± 0.07[a]	73.55 ± 1.86[c]	8.28 ± 0.016	96.93 ± 7.15
	Non-lipidated peptide 20	11.73 ± 0.06[c,e]	99.15 ± 1.39	7.43 ± 0.17	64.26 ± 3.97[d,e]	7.52 ± 0.23[e]	90.46 ± 10.65	11.54 ± 0.06 [f]	99.73 ± 1.41	7.39 ± 0.05	65.40 ± 1.04[d,e]	7.88 ± 0.13	96.93 ± 5.79
GCGR	Glucagon	12.46 ± 0.04	99.99 ± 0.93	6.43 ± 0.03	100.00 ± 1.71	7.69 ± 0.11	100.00 ± 5.39	11.33 ± 0.06	100.33 ± 1.66	6.22 ± 0.03	100.00 ± 1.74	7.28 ± 0.07	100.00 ± 3.91
	Peptide 20	11.80 ± 0.04[d]	100.21 ± 0.86	N.A.	N.A.	7.78 ± 0.18	119.72 ± 9.66	11.59 ± 0.06[a]	100.63 ± 1.36	N.A.	N.A.	7.26 ± 0.13	108.61 ± 6.40
	Non-lipidated peptide 20	8.62 ± 0.04[d,h]	103.07 ± 1.98	N.A.	N.A.	N.B.	N.B.	8.71 ± 0.05[d,h]	104.66 ± 2.16	N.A.	N.A.	N.B.	N.B.

cAMP accumulation and β-arrestin 2 recruitment assays were performed in HEK293T cells transiently transfected with wild-type GIPR, GLP-1R and GCGR constructs. Whole cell binding assay was performed in CHO-K1 cells transiently transfected with wild-type GIPR, GLP-1R and GCGR constructs. Dose-response curves were analyzed using a three-parameter logistic equation to obtain pEC_{50}, pIC_{50}, E_{max} and Span values. Data shown are means ± S.E.M. of at least three independent experiments (*n* = 3-4). One-way ANOVA and two-tailed Student's *t*-test were used to determine statistical difference. [a], P< 0.05, [b], P< 0.01, [c], P<0.001 and [d], P<0.0001 compared with endogenous ligands. [e], P< 0.05, [f], P< 0.01, [g], P<0.001 and [h], P<0.0001 compared between peptide 20 and non-lipidated peptide 20. N.A., not active. N.B., no binding was detected.

Summary of the data

Non-lipidated peptide 20 had poorer EC_{50} values for cAMP accumulation at GIPR (–871-fold) and GCGR (–1,514-fold) than peptide 20, but their potencies to GLP-1R were similar, consistent with that observed in the presence of 0.1% BSA. In presence of either 0.1% casein or 0.1% BSA, both peptides failed to recruit β-arrestin 2 mediated by GIPR and GCGR, while exhibiting similar β-arrestin 2 recruitment activities at GLP-1R. Non-lipidated peptide 20 was unable to compete with radiolabeled glucagon to bind GCGR regardless of 0.1% casein or 0.1% BSA, its binding abilities (IC_{50}) to GIPR and GLP-1R were decreased by 4- to 50-fold and 3- to 17-fold in the presence of 0.1% casein or 0.1% BSA, respectively.

Supplementary Table 11 | Signaling profiles of mono- and dual agonists at GIPR and GLP-1R

Receptor	Agonist	Casein						BSA					
		cAMP accumulation		β-arrestin 2 recruitment		Binding		cAMP accumulation		β-arrestin 2 recruitment		Binding	
		pEC_{50} ± S.E.M.	E_{max} ± S.E.M.	pEC_{50} ± S.E.M.	E_{max} ± S.E.M.	pIC_{50} ± S.E.M.	Span ± S.E.M.	pEC_{50} ± S.E.M	E_{max} ± S.E.M.	pEC_{50} ± S.E.M	E_{max} ± S.E.M.	pIC_{50} ± S.E.M.	Span ± S.E.M.
GIPR	GIP_{1-42}	12.21 ± 0.04	100.10 ± 1.15	8.01 ± 0.03	100.00 ± 1.10	8.61 ± 0.08	100.00 ± 3.47	11.82 ± 0.05	100.25 ± 1.55	7.59 ± 0.07	99.60 ± 2.31	7.85± 0.11	100.00 ± 4.96
	Tirzepatide	11.77 ± 0.07 [b]	99.96 ± 1.69	7.73 ± 0.11	53.41 ± 1.93[d]	8.50 ± 0.10	92.95 ± 3.97	10.90 ±0.05[d]	99.89 ± 1.35	6.20 ± 0.08[d]	53.44 ± 2.32[d]	7.00 ± 0.11[b]	99.58 ± 6.18
	Non-acylated tirzepatide	11.75 ± 0.03[b]	100.15 ± 0.77	7.83 ± 0.05	59.76 ± 1.01[d,e]	8.47 ± 0.13	88.80 ± 5.25	11.74 ± 0.07[h]	99.94 ± 1.51	7.19 ± 0.09[a,f]	57.08 ± 1.97[d]	7.88 ± 0.11[f]	89.06 ± 4.53
GLP-1R	$GLP\text{-}1_{(7\text{-}36)}NH_2$	12.53 ± 0.08	100.00 ± 1.83	7.79 ± 0.02	100.00 ± 0.68	7.88 ± 0.17	100.00 ± 7.81	11.58 ± 0.06	100.00 ± 1.40	7.31 ± 0.05	99.94 ± 1.83	8.09 ± 0.11	100.00 ± 5.06
	Tirzepatide	11.52 ± 0.07[d]	99.13 ± 1.73	N.A.	N.A.	7.51 ± 0.18	91.41 ± 8.36	9.99 ± 0.05[d]	99.70 ± 1.50	N.A.	N.A.	7.77 ± 0.18	84.12 ± 6.53
	Non-acylated tirzepatide	12.05 ± 0.04[b,f]	98.76 ± 0.97	7.56 ± 0.03[a]	40.77 ± 0.37[d]	7.61 ± 0.23	80.39 ± 9.18	11.33 ± 0.06[a,h]	99.14 ± 1.65	7.06 ± 0.04	36.60 ± 0.52[d]	7.92 ± 0.13	83.39 ± 4.46

cAMP accumulation and β-arrestin 2 recruitment assays were performed in HEK293T cells transiently transfected with wild-type GIPR and GLP-1R constructs. Whole cell binding assay was performed in CHO-K1 cells transiently transfected with wild-type GIPR and GLP-1R constructs. Dose-response curves were analyzed using a three-parameter logistic equation to obtain pEC_{50}, pIC_{50}, E_{max} and Span values. Data shown are means ± S.E.M. of at least three independent experiments (n = 3-5). One-way ANOVA and two-tailed Student's t-test were used to determine statistical difference. [a], $P< 0.05$, [b], $P< 0.01$, [c], $P<0.001$ and [d], $P<0.0001$ compared with endogenous ligands. [e], $P< 0.05$, [f], $P< 0.01$, [g], $P<0.001$ and [h], $P<0.0001$ compared between tirzepatide and non-acylated tirzepatide. N.A., not active.

Summary of the data

In the presence of 0.1% casein, tirzepatide and non-acylated tirzepatide induced similar levels of cAMP accumulation at GIPR, whereas in the presence of 0.1% BSA, the EC_{50} of non-acylated tirzepatide induced cAMP response was 6.92-fold higher than that of tirzepatide. For GLP-1R, non-acylated tirzepatide elicited a better cAMP response (+3.39-fold) compared to tirzepatide in the presence of 0.1% casein. However, such a difference is much more significant when 0.1% BSA is present (+21.88-fold). Similar phenomenon was observed in our β-arrestin 2 recruitment assay. While tirzepatide and non-acylated tirzepatide displayed similar β-arrestin 2 responses at GIPR in the presence of 0.1% casein, non-acylated tirzepatide showed a better efficacy in the presence of 0.1% BSA (+9.77-fold). In the case of GLP-1R, tirzepatide failed to induce β-arrestin 2 recruitment, whereas non-acylated tirzepatide did in the presence of either 0.1% casein or 0.1% BSA. In terms of competitive radiolabeled ligand binding to GIPR, there was no difference between tirzepatide and

non-acylated tirzepatide when 0.1% casein was present, but the binding of tirzepatide to GIPR was significantly reduced in the presence 0.1% BSA (−7.59-fold). No obvious difference between tirzepatide and non-acylated tirzepatide was observed for GLP-1R binding regardless of 0.1% casein or 0.1% BSA.

Supplementary Table 12 | Primers used in this study, related to Figs. 3, 5, Supplementary Figs. 2, 3, 4, 9 and Supplementary Tables 3, 6, 7, 8, 9, 10, 11

Oligonucleotide name	Oligonucleotide sequence (5'-3')	Cloning method	Product
Insert-GIPR(22-466)-forward	GACGGCAGCGCCGGCAGCGCCGGCAGCGCCAGGGCGGAGACAGGCTCTAAGGGGCAGACGGCG	Homologous recombination	pcDNA3.1-HA-GIPR(22-466) & pcDNA3.1-HA-BRIL-GIPR(22-421)(T345F)-15AA-LgBiT-2MBP
Insert-GIPR(22-466)-reverse	GCTGGATATCTGCAGAATTCTTAGCAGTAACTTTCCAACTCCCG		
Linear-pcDNA3.1-forward-1	GAATTCTGCAGATATCCAGC		
Linear-pcDNA3.1-reverse-1	GGCGCTGCCGGCGCTGCCGTCATCATCGTCCTTGTAGTC		
Insert-GIPR(structural construct)-forward	GACGGCAGCGCCGGCAGCGCCGGCAGCGCCGCTGATCTGGAAGACAATTGGGAAACTCTGAAC		
Insert-GIPR(structural construct)-reverse	GCTGGATATCTGCAGAATTCTTACTTGGTGATACGAGTCTGCGC		
Insert-GLP-1R(24-463)-forward	GCCGGCAGCGCCGGCAGCGCCCGCCCCCAGGGTGCCACTGTGTCC	Homologous recombination	pcDNA3.1-HA-GLP-1R(24-463) & pcDNA3.1-HA-GLP-1R(24-463)-15AA-LgBiT-2MBP
Insert-GLP-1R(24-463)-reverse	GACTCGAGCGGCCGCTTTTAGCTGCAGGAGGCCTGGCAAGTGGCTG		
Linear-pcDNA3.1-forward-2	TAAGAATTCTGCAGATATCCAGCACAGTG		
Linear-pcDNA3.1-reverse-2	GCGGGCGCTGCCGGCGCTGCCGGCGCTGCCGTC		
Insert-GLP-1R(structural construct)-forward	GCCGGCAGCGCCGGCAGCGCCCGCCCCCAGGGTGCCACTGTGTCC		
Insert-GLP-1R(structural construct)-reverse	GGATATCTGCAGAATTCTTACTTGGTGATACGAGTCTGCGCGTC		
Insert-GCGR(24-477)-forward	CTTCTGCCTGGTATTCGCCGGCGCGCCACAGGTGATGGACTTCCTGTTTGAGAAG	Homologous recombination	pcDNA3.1-HA-GCGR(27-477) & pcDNA3.1-HA-GCGR(27-432)-HPC4
Insert-GCGR(24-477)-reverse	GTGCTGGATATCTGCAGAATTCTCAGAAGGGGCTCTCAGCCAATCTAGGGAG		
Linear-pcDNA3.1-forward-3	TGAGAATTCTGCAGATATCCAGCACAGTGGCGG		
Linear-pcDNA3.1-reverse-3	GCCGGCGAATACCAGGCAGAAGATGTAG		
Insert-GCGR(structural construct)-forward	CATCTTCTGCCTGGTATTCGCCGGCGCGCCACAAGTGATGGATTTC		
Insert-GCGR(structural construct)-reverse	GTGCTGGATATCTGCAGAATTCTCATTTGCCATCGATCAGTCTGGGGTCCACC		
pcDNA3.1-GIPR-Rluc8-forward	TGTACAAAAAAGCAGGCTTCATGACTACCTCTCCGATCCTGCA	Homologous recombination	pcDNA3.1-GIPR-Rluc8
pcDNA3.1-GIPR-Rluc8-reverse	TTGTACAAGAAAGCTGGGTCGCAGTAACTTTCCAACTCCCGG		
Linear-pcDNA3.1-Rluc8-forward	GACCCAGCTTTCTTGTACAAAGTG		
Linear-pcDNA3.1-Rluc8-reverse	GAAGCCTGCTTTTTTGTACAAACTT		

pcDNA3.1-GLP-1R-Rluc8-forward	TGTACAAAAAAGCAGGCTTCATGGCCGGCGCCCCCGGC	Homologous recombination	pcDNA3.1-GLP-1R-Rluc8
pcDNA3.1-GLP-1R-Rluc8-reverse	TTGTACAAGAAAGCTGGGTCGCTGCTGGTGGGACACTTGA		
Linear-pcDNA3.1-Rluc8-forward	GACCCAGCTTTCTTGTACAAAGTG		
Linear-pcDNA3.1-Rluc8-reverse	GAAGCCTGCTTTTTTGTACAAACTT		
pcDNA3.1-GCGR-Rluc8-forward	TGTACAAAAAAGCAGGCTTCATGCCCCCCTGCCAGCCA	Homologous recombination	pcDNA3.1-GCGR-Rluc8
pcDNA3.1-GCGR-Rluc8-reverse	TTGTACAAGAAAGCTGGGTCGAAGGGGCTCTCAGCCAATC		
Linear-pcDNA3.1-Rluc8-forward	GACCCAGCTTTCTTGTACAAAGTG		
Linear-pcDNA3.1-Rluc8-reverse	GAAGCCTGCTTTTTTGTACAAACTT		
pcDNA3.1-Venus-β-arrestin 2-forward	CTTCGAATTCTGCAGTCGACATGGGTGAAAAACCCGGG	Homologous recombination	pcDNA3.1-Venus-β-arrestin 2
pcDNA3.1-Venus-β-arrestin 2-reverse	GGGCCCGCGGTACCAAGCTTCTAGCAGAACTGGTCATCACAGTCG		
Linear-pcDNA3.1-Venus-forward	AAGCTTGGTACCGCGGGC		
Linear-pcDNA3.1-Venus-reverse	GTCGACTGCAGAATTCGAAGC		
Q30A-forward	GACAGGCTCTAAGGGGGCGACGGCGGGGGAGCTG	Site-directed mutagenesis	pcDNA3.1-HA-GIPR(22-466)-Q30A
Q30A-reverse	CAGCTCCCCCGCCGTCGCCCCCTTAGAGCCTGTC		
Y36A-forward	GACGGCGGGGGAGCTGGCCCAGCGCTGGGAACGG		pcDNA3.1-HA-GIPR(22-466)- Y36A
Y36A-reverse	CCGTTCCCAGCGCTGGGCCAGCTCCCCCGCCGTC		
W90A-forward	CCCTGGTACCTGCCCGCGCACCACCATGTGGCTG		pcDNA3.1-HA-GIPR(22-466)- W90A
W90A-reverse	CAGCCACATGGTGGTGCGCGGGCAGGTACCAGGG		
F127A-forward	GAGAAGAATGAGGCCGCTCTGGACCAAAGGCTC		pcDNA3.1-HA-GIPR(22-466)- F127A
F127A-reverse	GAGCCTTTGGTCCAGAGCGGCCTCATTCTTCTC		
E135A-forward	CAAAGGCTCATCTTGGCGCGGTTGCAGGTCATG		pcDNA3.1-HA-GIPR(22-466)- E135A
E135A-reverse	CATGACCTGCAACCGCGCCAAGATGAGCCTTTG		
Y141A-forward	CGGTTGCAGGTCATGGCCACTGTCGGCTACTCC		pcDNA3.1-HA-GIPR(22-466)- Y141A
Y141A-reverse	GGAGTAGCCGACAGTGGCCATGACCTGCAACCG		
Y145A-forward	CAGCTTCCAGGTGATGGCCACAGTGGGCTACAGC		pcDNA3.1-HA-GIPR(22-466)- Y145A
Y145A-reverse	GCTGTAGCCCACTGTGGCCATCACCTGGAAGCTG		
R196A-forward	GACCGTCTGCTACCTGCACCTGGCCCCTACCTTG		pcDNA3.1-HA-GIPR(22-466)- R196A
R196A-reverse	CAAGGTAGGGGCCAGGTGCAGGTAGCAGACGGTC		

W209A-forward	CAGGCCCTTGCGCTGGCGAACCAGGCCCTCGCTG		pcDNA3.1-HA-GIPR(22-466)- W209A
W209A-reverse	CAGCGAGGGCCTGGTTCGCCAGCGCAAGGGCCTG		
Q285A-forward	CTGTACGAGAACACGGCGTGCTGGGAGCGCAAC		pcDNA3.1-HA-GIPR(22-466)- Q285A
Q285A-reverse	GTTGCGCTCCCAGCACGCCGTGTTCTCGTACAG		
T345F-forward	GCTCGCTCCACGCTGTTTCTGGTGCCCCTGCTG		pcDNA3.1-HA-GIPR(22-466)- T345F
T345F-reverse	CAGCAGGGGCACCAGAAACAGCGTGGAGCGAGC		
R370A-forward	GCCCGGGGCGCCCTGGCCTTCGCCAAGCTCGGC		pcDNA3.1-HA-GIPR(22-466)- R370A
R370A-reverse	GCCGAGCTTGGCGAAGGCCAGGGCGCCCCGGGC		
D191A-forward	GCCATTCTCAGCCGAGCCCGTCTGCTACCTCGAC		pcDNA3.1-HA-GIPR(22-466)- D191A
D191A-reverse	GTCGAGGTAGCAGACGGGCTCGGCTGAGAATGGC		
R192A-forward	CATTCTCAGCCGAGACGCTCTGCTACCTCGACCTG		pcDNA3.1-HA-GIPR(22-466)- R192A
R192A-reverse	CAGGTCGAGGTAGCAGAGCGTCTCGGCTGAGAATG		
Q138A-forward	CATCTTGGAGCGGTTGGCGGTCATGTACACTGTC		pcDNA3.1-HA-GIPR(22-466)- Q138A/D191A/R196A
Q138A-reverse	GACAGTGTACATGACCGCCAACCGCTCCAAGATG		
W39A-forward	GAGACGGTGCAGAAAGCGCGAGAATACCGACGC	Site-directed mutagenesis	pcDNA3.1-GLP-1R(1-30)-Flag-GLP-1R(31-463)-W39A
W39A-reverse	GCGTCGGTATTCTCGCGCTTTCTGCACCGTCTC		
W91A-forward	CCCTGGTACCTGCCCGCGGCCAGCAGTGTGCCG		pcDNA3.1-GLP-1R(1-30)-Flag-GLP-1R(31-463)-W91A
W91A-reverse	CGGCACACTGCTGGCCGCGGGCAGGTACCAGGG		
R134A-forward	GTCCAAGCGAGGGGAAGCAAGCTCCCCGGAGGAG		pcDNA3.1-GLP-1R(1-30)-Flag-GLP-1R(31-463)-R134A
R134A-reverse	CTCCTCCGGGGAGCTTGCTTCCCCTCGCTTGGAC		
Y148A-forward	GTTCCTCTACATCATCGCCACGGTGGGCTACGCAC		pcDNA3.1-GLP-1R(1-30)-Flag-GLP-1R(31-463)-Y148A
Y148A-reverse	GTGCGTAGCCCACCGTGGCGATGATGTAGAGGAAC		
Y205A-forward	GCCCTGAAGTGGATGGCTAGCACAGCCGCCCAG		pcDNA3.1-GLP-1R(1-30)-Flag-GLP-1R(31-463)-Y205A
Y205A-reverse	CTGGGCGGCTGTGCTAGCCATCCACTTCAGGGC		
Q210A-forward	GTATAGCACAGCCGCCGCGCAGCACCAGTGGGATG		pcDNA3.1-GLP-1R(1-30)-Flag-GLP-1R(31-463)-Q210A
Q210A-reverse	CATCCCACTGGTGCTGCGCGGCGGCTGTGCTATAC		
Y152A-forward	CATCTACACGGTGGGCGCCGCACTCTCCTTCTCTG		pcDNA3.1-GLP-1R(1-30)-Flag-GLP-1R(31-463)-Y152A
Y152A-reverse	CAGAGAAGGAGAGTGCGGCGCCCACCGTGTAGATG		

W214A-forward	GCCCAGCAGCACCAGGCGGATGGGCTCCTCTCC		pcDNA3.1-GLP-1R(1-30)-Flag-GLP-1R(31-463)-W214A
W214A-reverse	GGAGAGGAGCCCATCCGCCTGGTGCTGCTGGGC		
D198A-forward	CATTGTCCGTCTTCATCAAGGCCGCAGCCCTGAAGTGG		pcDNA3.1-GLP-1R(1-30)-Flag-GLP-1R(31-463)-D198A
D198A-reverse	CCACTTCAGGGCTGCGGCCTTGATGAAGACGGACAATG		
K202A-forward	CAAGGCCGCAGCCCTGGCGTGGATGTATAGCACAG		pcDNA3.1-GLP-1R(1-30)-Flag-GLP-1R(31-463)-K202A
K202A-reverse	CTGTGCTATACATCCACGCCAGGGCTGCGGCCTTG		
R190A-forward	GCATCCTTCATCCTGGCAGCATTGTCCGTCTTC		pcDNA3.1-GLP-1R(1-30)-Flag-GLP-1R(31-463)-R190A
R190A-reverse	GAAGACGGACAATGCTGCCAGGATGAAGGATGC		
K197A-forward	CATTGTCCGTCTTCATCGCGGACGCAGCCCTGAAGTG		pcDNA3.1-GLP-1R(1-30)-Flag-GLP-1R(31-463)-K197A
K197A-reverse	CACTTCAGGGCTGCGTCCGCGATGAAGACGGACAATG		
R299A-forward	CGAGGGCTGCTGGACCGCGAACTCCAACATGAAC		pcDNA3.1-GLP-1R(1-30)-Flag-GLP-1R(31-463)-R299A
R299A-reverse	GTTCATGTTGGAGTTCGCGGTCCAGCAGCCCTCG		
N300A-forward	GGCTGCTGGACCAGGGCCTCCAACATGAACTAC		pcDNA3.1-GLP-1R(1-30)-Flag-GLP-1R(31-463)-N300A
N300A-reverse	GTAGTTCATGTTGGAGGCCCTGGTCCAGCAGCC		
Y145A-forward	CAGCTCCTGTTCCTCGCCATCATCTACACGGTG		pcDNA3.1-GLP-1R(1-30)-Flag-GLP-1R(31-463)-Y145A/D198A/K202A
Y145A-reverse	CACCGTGTAGATGATGGCGAGGAACAGGAGCTG		
W87A-forward	CCCTGGTACCTGCCTGCGCACCACAAAGTGCAAC	Site-directed mutagenesis	pcDNA3.1-GCGR(1-477)-W87A-V5-6×His
W87A-reverse	GTTGCACTTTGTGGTGCGCAGGCAGGTACCAGGG		
Q131A-forward	GAGGAGATTGAGGTCGCGAAGGAGGTGGCCAAG		pcDNA3.1-GCGR(1-477)-Q131A-V5-6×His
Q131A-reverse	CTTGGCCACCTCCTTCGCGACCTCAATCTCCTC		
Y138A-forward	GAGGTGGCCAAGATGGCCAGCAGCTTCCAGGTG		pcDNA3.1-GCGR(1-477)-Y138A-V5-6×His
Y138A-reverse	CACCTGGAAGCTGCTGGCCATCTTGGCCACCTC		
Y145A-forward-1	CAGCTTCCAGGTGATGGCCACAGTGGGCTACAGC		pcDNA3.1-GCGR(1-477)-Y145A-V5-6×His
Y145A-reverse-1	GCTGTAGCCCACTGTGGCCATCACCTGGAAGCTG		
Y202A-forward	CTGCTCAGGACCCGCGCCAGCCAGAAAATTGGC		pcDNA3.1-GCGR(1-477)-Y202A-V5-6×His
Y202A-reverse	GCCAATTTTCTGGCTGGCGCGGGTCCTGAGCAG		

K205A-forward	GACCCGCTACAGCCAGGCAATTGGCGACGACCTC		pcDNA3.1-GCGR(1-477)-K205A-V5-6×His
K205A-reverse	GAGGTCGTCGCCAATTGCCTGGCTGTAGCGGGTC		
W215A-forward	CTCAGTGTCAGCACCGCGCTCAGTGATGGAGCG		pcDNA3.1-GCGR(1-477)-W215A-V5-6×His
W215A-reverse	CGCTCCATCACTGAGCGCGGTGCTGACACTGAG		
Q293A-forward	CTGTTCGAGAACGTCGCGTGCTGGACCAGCAATG		pcDNA3.1-GCGR(1-477)-Q293A-V5-6×His
Q293A-reverse	CATTGCTGGTCCAGCACGCGACGTTCTCGAACAG		
D195A-forward	CTCCGTGCTGGTCATTGCTGGGCTGCTCAGGACC		pcDNA3.1-GCGR(1-477)-D195A-V5-6×His
D195A-reverse	GGTCCTGAGCAGCCCAGCAATGACCAGCACGGAG		
R199A-forward	CATTGATGGGCTGCTCGCGACCCGCTACAGCCAG		pcDNA3.1-GCGR(1-477)-R199A-V5-6×His
R199A-reverse	CTGGCTGTAGCGGGTCGCGAGCAGCCCATCAATG		
Q142A-forward	GATGTACAGCAGCTTCGCGGTGATGTACACAGTG		pcDNA3.1-GCGR(1-477)-Q142A/D195A/R199A-V5-6×His
Q142A-reverse	CACTGTGTACATCACCGCGAAGCTGCTGTACATC		

References

1 Sanchez-Garrido, M. A. *et al.* GLP-1/glucagon receptor co-agonism for treatment of obesity. *Diabetologia* **60**, 1851-1861, doi:10.1007/s00125-017-4354-8 (2017).

2 Williams, D. M., Nawaz, A. & Evans, M. Drug Therapy in Obesity: A Review of Current and Emerging Treatments. *Diabetes Ther* **11**, 1199-1216, doi:10.1007/s13300-020-00816-y (2020).

3 Brandt, S. J., Götz, A., Tschöp, M. H. & Müller, T. D. Gut hormone polyagonists for the treatment of type 2 diabetes. *Peptides* **100**, 190-201, doi:10.1016/j.peptides.2017.12.021 (2018).

4 Yang, D. *et al.* G protein-coupled receptors: structure- and function-based drug discovery. *Signal Transduct Target Ther* **6**, 7, doi:10.1038/s41392-020-00435-w (2021).

5 Tschop, M. H. *et al.* Unimolecular Polypharmacy for Treatment of Diabetes and Obesity. *Cell Metab* **24**, 51-62, doi:10.1016/j.cmet.2016.06.021 (2016).

6 Usui, R., Yabe, D. & Seino, Y. Twincretin as a potential therapeutic for the management of type 2 diabetes with obesity. *J Diabetes Investig* **10**, 902-905, doi:10.1111/jdi.13005 (2019).

7 Svegliati-Baroni, G., Patricio, B., Lioci, G., Macedo, M. P. & Gastaldelli, A. Gut-Pancreas-Liver Axis as a Target for Treatment of NAFLD/NASH. *Int J Mol Sci* **21**, 5820, doi:10.3390/ijms21165820 (2020).

8 Bluher, M. Metabolically Healthy Obesity. *Endocr Rev* **41**, 405–420, doi:10.1210/endrev/bnaa004 (2020).
